# Exploring the anti-cancer potential of *Ganoderma lucidum* polysaccharides (GLPs) and their versatile role in enhancing drug delivery systems: a multifaceted approach to combat cancer

**DOI:** 10.1186/s12935-023-03146-8

**Published:** 2023-12-16

**Authors:** Xiaoli Gao, Mina Homayoonfal

**Affiliations:** 1 Department of Life Science, Lyuliang University, Lyuliang, 033001 Shanxi China; 2 Research Center for Biochemistry and Nutrition in Metabolic Diseases, Institute for Basic Sciences, Kashan University of Medical Sciences, Kashan, I.R. of Iran

**Keywords:** *Ganoderma lucidumm*, Polysaccharides, Cancer, Nanoparticle, Immunoregulation

## Abstract

There has been a growing global interest in the potential health benefits of edible natural bioactive products in recent years. *Ganoderma lucidum*, a medicinal mushroom, has gained attention for its decadent array of therapeutic and pharmaceutical compounds. Notably, *G. lucidum* exhibits significant anti-cancer effects against various cancer types. Polysaccharides, a prominent component in *G. lucidum*, are pivotal in conferring its diverse biological and medicinal properties. The primary focus of this study was to investigate the anti-cancer activities of *G. lucidum* polysaccharides (GLPs), with particular attention to their potential to mitigate chemotherapy-associated toxicity and enhance targeted drug delivery. Our findings reveal that GLPs exhibit anti-cancer effects through diverse mechanisms, including cytotoxicity, antioxidative properties, apoptosis induction, reactive oxygen species (ROS) generation, and anti-proliferative effects. Furthermore, the potential of GLPs-based nanoparticles (NPs) as delivery vehicles for bioactive constituents was explored. These GLPs-based NPs are designed to target various cancer tissues, enhancing the biological activity of encapsulated compounds. As such, GLPs derived from *G. lucidum* represent a promising avenue for inhibiting cancer progression, minimizing chemotherapy-related side effects, and supporting their utilization in combination therapies as natural adjuncts.

## Introduction

Mushrooms have long been recognized in traditional medicine for their therapeutic potential. However, enhancing their bioavailability and therapeutic efficacy in human health remains challenging [96]. Despite significant advancements in medical care and products, there is an increasing demand for natural therapeutic agents with minimal adverse effects [10, 74].

In recent decades, various types of mushrooms have been explored for their therapeutic and health-promoting properties [73]. Medicinal mushrooms, including Ganoderma, have demonstrated a wide range of health benefits, such as anti-cancer, anti-immunomodulatory, anti-inflammatory, antimicrobial, and antioxidant effects, which can be attributed to their bioactive compounds, including alkaloids, lanostanoids, lectins, phenolic compounds, polysaccharides (β-glucan), polysaccharides-peptide and –protein complexes, sterols, and terpenoids [5, 94, 133–135]. As a result, there has been a surge of interest in developing novel therapeutic agents derived from mycelium or medicinal mushroom fruit bodies.

*Ganoderma*, a genus of polypore fungi found mainly in tropical regions, comprises around 80 species [78]. These fungi are characterized by their bitter, fanlike, rigid, or hoof-like appearance. The classification of *Ganoderma* species has been challenging due to morphological variations, synonyms, and incorrect naming practices [105]. However, DNA sequence data have enabled the categorization of *Ganoderma* species into monophyletic genera, including *G. applanatum*, *G. colossus*, *G. tsugae*, *Asian G. lucidum*, *G. meredithiae*, and *G. resinaceum* [35]. *G. lucidum*, also known as "Lingzhi" in China and "Reishi" or "Manetake" in Japan, is an edible fungus with a history spanning over two centuries in Asian countries, where it has been revered for its potential to promote health and longevity. *G. lucidum* offers a comprehensive range of pharmacological and therapeutic features, such as antibacterial, anti-fibrotic, anti-HIV, antioxidant, anti-tumor, hypoglycemic, immunoregulatory, anti-angiogenic, liver-protective, cholesterol-lowering, and lower urinary tract symptom-reducing activities [2].

Extensive research has shown that these therapeutic effects are closely associated with a variety of chemicals found in *G. lucidum*, including alkaloids, amino acids, enzymes, flavonoids, minerals, polysaccharides, proteins, steroids, triterpenes, and vitamins [1, 4]. Polysaccharides and triterpenoid compounds from G. lucidum have recently gained considerable attention in medical research, primarily due to their significant pharmacological attributes, particularly their anti-cancer effects. *G. lucidum* yields various metabolites, with more than 341 secondary metabolites, 380 terpenoids, and 200 polysaccharides identified from its fruit bodies, mycelia, and spores. Among these, (1 → 3), (1 → 6) α/β-D-glucans, protein-polysaccharide complexes, and water-soluble heteropolysaccharides are the principal bioactive components [42, 49, 78]. Notably, glycoproteins and heteropolysaccharides consist of different ratios of arabinose, fucose, glucose, galactose, mannose, and xylose connected by various glycosidic linkages and peptide bonds [15, 114]. The highly complex nature of polysaccharides necessitates a thorough examination of their properties, including glycosidic bonds, molecular weight, and sugar components, to elucidate structure-biological activity relationships.

Furthermore, the molecular properties of GLPs have rendered them functional biomaterials, amenable to conjugation or cross-linking with active compounds. This modification imparts stimuli-responsiveness, enabling them to target tumors and sustain the controlled release of cargo in the tumor microenvironment, characterized by oxidative and acidic conditions. These engineered derivatives of GLPs exhibit reduced viscosity while retaining excellent biocompatibility and biodegradability [122]. The development of polysaccharide-based nanocarriers and adjuvants has gained prominence to augment the health benefits of polysaccharides further and broaden their applications. Research has highlighted the remarkable functional properties and processing advantages of polysaccharide-based nanoparticulate co-delivery systems, particularly in the functional food and pharmaceutical industries [7]. These nanocarriers can encapsulate, protect, and precisely target the delivery of various bioactive substances, ensuring controlled release within complex physiological microenvironments. Another focus of current research is the co-delivery of drugs or active ingredients using GLPs as carriers. Notably, when incorporated into nanoparticles, GLPs retain their intrinsic functions, such as immunomodulation and antitumor activity, and play a vital role in preventing the rapid metabolism of other active components. This enhances loading capacity, encapsulation efficiency, and, ultimately, the bioactivity and pharmacological effects of polysaccharide-based nano-systems [17, 91].

In the current study, a fresh and innovative perspective on the anticancer potential of *G. lucidum* and its bioactive constituents, particularly polysaccharides, is offered, building upon the foundations laid by previous research. Acknowledgment is given to seminal works conducted by Wang and Yu [116], Ahmad [2], Gill, Navgeet et al. (2017), Sohretoglu and Huang [106], and Kladar, Gavaric et al. [51], which have contributed to the understanding of the anticancer potential of *G. lucidum*, especially its polysaccharides. However, in our study, specific gaps left unexplored are addressed. Notably, the feasibility of utilizing GLPs as nanoparticle (NP) carriers for targeted drug delivery is introduced as an innovative facet of this field. This approach capitalizes on the unique properties of GLPs, such as their biocompatibility and low toxicity, to enhance the delivery of bioactive compounds. By targeting various cancer tissues, these GLPs-based NPs aim to improve the efficacy of cancer treatment.

Furthermore, the mechanisms underlying the anti-cancer effects of GLPs, encompassing cytotoxicity, antioxidative properties, apoptosis induction, reactive oxygen species generation, and anti-proliferative effects, are investigated by our work. Moreover, the present study addresses the issue of chemotherapy-associated toxicity and seeks to minimize these side effects by utilizing GLPs-based NPs. The innovative aspect of our work also lies in its potential for application in combination therapies. By highlighting the versatility of GLPs as natural adjuncts in combination with other therapeutic agents, our research takes a step further toward offering a comprehensive approach to cancer treatment. In this regard, we searched systematically online databases including: PubMed-Medline, Embase, ISI Web of Science and Cochrane Central Register of Controlled Trials until October, 2023. Search words, including Mesh and text words, were applied as followed: “Ganoderma lucidumm “ and “polysaccharides “, and “, “cancer’ and “nanoparticle” and “immunoregulation”.

## The polysaccharides characterizations of *G. lucidum*

Various polysaccharides extracted from *G. lucidum* are classified by their level of branching, higher or tertiary structures, molecular weight, and composition **(**Table 1**)**. Different GLPs are formed from complexes of homo-glucans, hetero-β-glucans, heteroglycans, and α-manno-β-glucan compositions [23, 90]. Homoglucans are biopolymers with linear or branched structures owning a backbone consisting of α- or β-linked glucose monomers such as (1 → 6)-β-glucans and (1 → 6)-α-glucans. Homoglucans can have side chains joined to the polymer structures at different sites [41]. β-glucan, known as the main constituents of the cell walls of higher fungi, is a glucose polymer that may be found as a linear structure with (1 → 3)-β-glucans backbone or branched ones with the same system containing (1 → 6)-β-glucans branches [11, 31]. Hetero-glucans encompass arabinose, galactose, glucuronic acid, mannose, ribose, and xylose as the prime constituents in different compositions [19]. Glycans are another polysaccharide isolated from *G. lucidum*, owning other monosaccharides than glucose in their backbone structure. They are categorized based on the monosaccharides of the backbone, such as fucans, galactan, mannans, and xylan (Stiger-Pouvreau, Bourgougnon et al. 2016, [32]. The design of hetero-glycans, regardless of being linear or branched, contains several monosaccharides, including arabinose, fucose, galactose, glucose, glucuronic acid, mannose, and xylose, as the main components or in composition [45].

**Table 1:** A summary of the monosaccharide composition of different GLPs applied in various studies

GLPs source	Monosaccharides composition	Molecular weight	Refs.
Fruiting bodies	D-Glu, D-Gal, D-Man, L- (or D)-Ara, D-Xyl, and L-Fuc	0.8 ~ 1 × 10^5^ Da	[107]
Spore	Backbone: D-Glu Branch: D-Glu	1.40 × 10^4^ Da	[6]
Submerged culture broth of *G. lucidum*	Backbone: D-Gal Branch: L-Arb, D-Glu, D- Man, D-Rha	2.2 × 10^4^ Da	[62]
Fruiting bodies	Backbone: D-Gal, D-Glu Branch: L-Fuc	1.2 × 10^4^ Da	[128, 130]
Fruiting bodies	Backbone: D-Gal, D-Glu Branch: L-Fuc	2.8 × 10^4^ Da	[128, 130]
Fruiting bodies	LZ-D-4: L-Ara, L-Fuc, D-Gal, D-Glu, D- Man, D-Xyl LZ-D-9: L-Fuc, D-Gal, D-Glu, D- Man	LZ-D-4: 1.56 × 10^4^ Da LZ-D-4: 1.30 × 10^4^ Da	[127, 129]
Fruiting bodies	Backbone: D-Gal, D-Glu Branch: D-Glu, L-Fuc	7 × 10^3^ Da	[127, 129]
Fruiting bodies	GLP-1: D-Glu GLP-2: D-Glu	GLP-1: 5.2 × 10^3^ Da GLP-2: 1.54 × 10^4^ Da	[67]
Fruiting bodies	Backbone: D-Gal, D-Glu Branch: D-Glu, L-Fuc	1.12 × 10^4^ Da	[126]
Fruiting bodies	Backbone: D-Gal, D-Glu Branch: D-Gal, D-Glu	2.5 × 10^6^ Da	[40]
Spore	Backbone: D-Glu Branch: D-Glu	1.03 × 10^5^ Da	[20]
Fruiting bodies	D-Glu	3.979 × 10^3^ Da	[50]
Fruiting bodies	Backbone: D-Glu Branch: D-Gal	3.75 × 10^6^ Da	[70]
Fruiting bodies	Backbone: D-Gal, D-Glu, D-GluA, L-Rha Branch: D-Glu	1.002 × 10^5^ Da	[82]
Spore	D-Glu	1.579 × 10^5^ Da	[117]
Spore	Backbone: D-Glu Branch: D-Glu	1.5 × 10^4^ Da	[25]
Fruiting bodies	GLP_HWE_: L-Ara, D-Gal, D-Glu, D- Man, D-Rha GLP_UAE_: L-Ara, D-Gal, D-Glu, D- Man, D-Rha	GLP_HWE_: 7.03 × 10^5^ Da GLP_UAE_: 4.65 × 10^5^ Da	[48]
Fruiting bodies	GLP: D-Fru, D-Gal, D-GalA, D-Glu, D-GluA, D- Man, D-Rha, D-Xyl Degraded GLP: D-Fru, D-Gal, D-GalA, D-Glu, D-GluA, D- Man, D-Rha, D-Xyl	GLP: 3.06 × 10^6^ Da Degraded GLP: 1.36 × 10^4^ Da	[123]
Fruiting bodies	GLP-1: D-Gal, D-Glu GLP-2: D-Glu	GLP-1: 1.072 × 10^5^ Da GLP-2: 1.95 × 10^4^ Da	[58]
Spore	Backbone: D-Glu Branch: D-Glu	1.93 × 10^5^ Da	[66, 69]
Fruiting bodies	D-Gal, D-Glu, D- Man, D-Xyl, L-Fuc	2.05 × 10^4^ Da	[12]
Spore	Backbone: D-Glu Branch: D-Glu	1.28 × 10^5^ Da	[99]
Spore	L-Ara, D-Gal, D-Glu	8.2 × 10^4^ Da	[118]

Arabinose (Ara), Fructose (Fru), Galactose (Gal), Galacturonic acid (GalA), Glucose (Glu), Glucuronic acid (GluA), Mannose (Man), Rhamnose (Rha), Xylose (Xyl), HWE: hot water extraction, UAE: ultrasound assisted extraction

Polysaccharides may link to various proteins and peptides through covalent bonds to form protein- or peptide-polysaccharide structures. Glycoproteins are carbohydrate-protein complexes found in compounds such as α-glucan-protein, β-glucan-protein, and heteroglycan-protein complexes. The design of glycopeptides is similar to glycoproteins with shorter amino acid chains [75], Zhao, He et al. [139]). Furthermore, proteoglycans are a type of glycoprotein with a highly glycosylated degree. Their structure comprises a core protein owning one or more covalently linked glycosaminoglycan chain(s) [144, 145]. Polysaccharides have received significant attention considering their variation and abundance in the body of *G. lucidum* and enjoying enormous biological potential. However, remarkable variations in the chemical structure of such polysaccharides and compositions result in massive diversity in their bioactivity effects. Hence, identifying the relationship between structural characteristics and biological activities is paramount.

## The association between structures of GLPs and their bioactivity

As mentioned, GLPs are physiologically active compounds generally called biological response modifiers (BRMs). Recently, in the investigation into the relationship between structural and biological properties of GLPs, the focus of attention has been on the activities of their BRMs [92]. Accordingly, various studies have evaluated the relation between the structural properties of GLPs, namely molecular weight, monosaccharides constitutions, the levels of glycosidic linkage, the configuration of linear and branch chains, and the class and contents of substituents.

### Tertiary structure: a key to enhanced bioactivity

GLPs with a tertiary structure show increased biological activity. GLPs may have a tertiary configuration as a triple helix, indicating the overall structure of these macromolecules. The tertiary structure occurs by forming hydrogen bonds between the second carbon positions and strengthening by side chains. It is assumed that the biological functions of GLPs are mostly related to their β-structures (tertiary conformation) [85]. It has been reported that a high molecular weight, triple helix structure of β-D-glucan is available in *G. lucidum.* The ordered β-structure is dissociated and converted to a random coil in harsh environments such as alkaline, carbamide, and dimethyl sulfoxide. This conformational transition reduces the bioactivities of GLPs [76]. The triple helical structure of (1 → 3)-β-glucan molecules is essential for their immune-modulating and anti-cancer potential, but underlying mechanisms remain unclear [136].

### Molecular weight: controversies and impacts

Molecular weight is a bioactivity-associated parameter that contradicts ideas that have been stated about its biological effects. While various studies have disclosed that the higher molecular weight of GLPs results in increased bioactivities, others have revealed the opposite relation [76, 123]. Polysaccharides with high molecular weights are typically linked to heightened immunomodulatory effects. They also play a crucial role in maintaining gastrointestinal equilibrium. However, it is worth noting that their solubility tends to decrease [59, 75, 100]. These high molecular weight polysaccharides can create many active polymer structures and initiate robust cascade signals through efficient receptor recognition patterns, thus activating the immune system effectively. Furthermore, these polysaccharides exhibit prebiotic qualities utilized by microorganisms within the host's digestive system. This utilization has been demonstrated to significantly enhance various aspects of intestinal barrier function, including physical, chemical, immune, and biological defenses, ultimately improving overall immune capacity. Additionally, they have shown potential in reducing blood sugar levels and enhancing antioxidant capabilities, contributing to the overall well-being of the host [133–135].

Conversely, lower molecular weight polysaccharides are known to facilitate improved in vivo digestion and absorption [67]. However, they tend to lack the ability to form active polymeric structures, which can hinder their interaction with proteins or cell surface receptors [47]. Similarly, polysaccharides with optimal branching (within the range of 0.2 to 1.3) can effectively trigger host immune responses by binding to specific cellular receptors. Yet, excess branches can lead to reduced water solubility and fewer recognition sites for host receptors, subsequently diminishing their bioactivity [125].

Furthermore, high molecular weight *G. lucidum* polysaccharides (3978 kDa), composed of fucose, mannose, glucose, and galactose and linked by β-(1 → 3)-Glu and β-(1 → 3, 6)-Glu residues, exhibited superior immunomodulatory activity compared to low molecular weight variants [59]. On the other hand, *G. lucidum* polysaccharides with a molecular weight of 1000 kDa have been shown to significantly inhibit the phosphorylation of FAK, AKT, and Smad2 and induce TGFβR and EGFR degradation in both in vivo and in vitro settings, effectively suppressing lung cancer cell growth and migration [102]. However, it is crucial to note that the activity and functionality of polysaccharides cannot solely be dependent on higher molecular weight. In contrast, specific polysaccharides with relatively lower molecular weight also demonstrate favorable immune activity.

### Degree of backbone branching: a balancing act

The degree of backbone branching is another effective parameter of the functional roles of GLPs. When the number of monosaccharides in branched backbones of GLPs exceeds four, they indicate no anti-tumor effects. Furthermore, when the substitution (branching) level is about 20–33%, the biological activity is high [53, 124]. Some chemical modifications such as acetylation, alkylation, carboxymethylation, and sulfation may be applied to alter the functional substitutions. Such changes generally improve the water solubility and, therefore, the bioactivity of GLPs [122]. Although greater molecular weight, lower branching degree, and higher water solubility directly correlate with anti-cancer effects, molecules with such properties should first have the primary glycosidic bonds essential for anti-cancer responses [23].

### Monosaccharide composition: a bioactive diversity

The primary focus of the structural characterization of GLP revolves around determining its sugar content, molecular weight (MW), the composition of monosaccharides, their ratios, the arrangement of sugar chains, the glycosidic linkages employed, and, in more extensive investigations, the three-dimensional spatial structure of GLP. GLP is a complex mixture of various monosaccharides (Table 1), which endows it with diverse structural and biological characteristics depending on the relative proportions and overall content of these monosaccharides. In particular, GLP is predominantly comprised of D-glucose (D-Glc), D-fructose (D-Fru), D-galactose (D-Gal), D-mannose (D-Man), D-xylose (D-Xyl), L-fucose (L-Fuc), L-rhamnose (L-Rha), L-arabinose (L-Ara) (Liu, Yang et al. 2022). Furthermore, it is essential to note that the monosaccharide composition and proportion of polysaccharides derived from different source parts can vary significantly. It has been demonstrated that GLPs with different monosaccharide compositions show different bioactive potentials. For instance, GLPs with high levels of galactose and rhamnose have anti-inflammatory and antioxidant activity, respectively [14, 133–135]. It seems that the anti-tumor activity of GLPs containing glucose and mannose is associated with their immune-simulating effects since a polysaccharide receptor with high specificities for glucose and mannose exists in human macrophages [41].

Polysaccharides derived from *G. lucidum* have the potential to mitigate the progressive risk of cancer due to their content of glucose and mannose. These specific monosaccharides can be recognized and primarily mediated by specific cell surface receptors. These receptors include dectin-1, the mannose receptor (MR), toll-like receptor (TLR) 4, complement receptor type 3 (CR3), scavenger receptors, and TLR2, which are present on the surface of effector cells [37]. To analyze the interactions between carbohydrates and immune receptors, Hsu, Cheng et al. [37], conducted experiments that showed interactions between dectin-1, TLR2, TLR4, and the macrophage mannose receptor (MMR) with the polysaccharide extracts from *G. lucidum*.

TLRs (Toll-like receptors) are transmembrane glycoproteins expressed at the cell surface or within endosomes. They are categorized as type I transmembrane glycoproteins and are part of the pattern recognition receptor (PRR) family. TLRs play a crucial role as components of the innate immune defense system in humans [24]. Interestingly, it was observed that mannan had a higher affinity for TLR2 and TLR4 compared to mannose. This suggests that the number of epitopes present in the tertiary structure of polysaccharides impacts the recognition of TLRs [8].

CR3 is a transmembrane glycoprotein composed of CD11b, non-covalently associated with CD18. It is expressed widely on various immune cells such as neutrophils, monocytes, and natural killer (NK) cells. CR3 acts as a receptor for both complement proteins and β-glucan. On the other hand, the MR belongs to the C-type lectin-like receptor family and is responsible for recognizing glycosylated molecules containing either a mannose residue or an N-acetyl glucose residue [61]. When effector cells encounter polysaccharides derived from *G. lucidum* in the peripheral blood, these receptors are pivotal in initiating the immune response. This activation prompts the effector cells to release cytokines, which, in turn, contribute to the host's ability to mount a robust and intensive immune response [91].

### Homopolysaccharides vs. Heteropolysaccharides: diverse anti-tumor properties

Polysaccharides derived from *G. lucidum* come in two distinct forms: homopolysaccharides and heteropolysaccharides [90]. Homopolysaccharides have demonstrated the ability to inhibit the growth of tumor cells by suppressing cyclin production and triggering cellular stress responses [119]. On the other hand, heteropolysaccharides obtained from *G. lucidum* have also shown remarkable anti-tumor and immunomodulatory properties. These properties can be attributed to the diverse array of monosaccharides, such as mannose, xylose, fucose, glucose, galactose, and arabinose, which are major components or occur in various combinations within these heteropolysaccharides [103]. Proteoglycans are highly branched heteropolysaccharides covalently attached to a protein. Serine and threonine residues are bonded to GLPs via O-glycosidic bonds, broken via β-carbon elimination, leading to reduced bioactivity of GLPs. These findings indicate that protein branches augment the functional impacts of GLPs [81]. Furthermore, high levels of acidic heteropolysaccharides, rich in uronic acid, enhance antioxidant and immunomodulatory activities compared to homopolysaccharides. However, the precise mechanisms responsible for these effects are yet to be fully elucidated [28, 55–57]. Most glycosidic bonds in GLPs consist of β-(1 → 3)-glucans, which exhibit notable immune-enhancing and antitumor properties (Kou, Ge et al. 2023).

### The role of specific glycosidic bonds: essential for bioactivity

It is important to note that polysaccharides containing β-glucan or β-(1 → 3)-glucan are more effective in their anti-tumor and immune-enhancing functions than those polysaccharides containing α-glucan and β-(1 → 6)-glucan [9, 112]. Accordingly, β-glucan molecules that (1 → 6)-β-linkage is their central structural bonds illustrate low anti-cancer activity, probably owing to their inherent flexibility of containing too various conformations [136].

The proposition suggests that polysaccharides consisting exclusively of β-(1 → 6)-glucan are not acknowledged by pattern recognition receptors (PRRs), such as Dectin-1, which typically recognize β-glucan and β-(1 → 3) [80]. Consequently, such polysaccharides either go unrecognized or display inferior biological properties. It is worth highlighting that most GLPs are primarily composed of a primary linear β-(1 → 3)-D-glucan chain. These chains feature β-(1 → 6)-glucose short-branch chains that can bind to cell receptors. This binding, in turn, triggers both innate and adaptive immune responses. Additionally, these polysaccharides exhibit the capacity to hinder tumor growth, although their mechanism of action leans towards inhibiting tumor growth rather than directly killing cancer cells. This aligns with the pertinent research findings indicating that β-(1 → 3) and β-(1 → 3)/(1 → 6)-glucan have significant potential for modulating immune responses, thus reducing the risk of tumor development through the recognition of pattern receptors. In contrast, β-(1 → 6) and (1 → 4) exhibit relatively lower immune activity but possess appropriate antioxidant capabilities.

### Future directions: the promise of customized therapies

Our exploration of the intricate relationship between the structural properties of GLPs and their bioactivity reveals the profound potential of these compounds in biomedical research and clinical applications. The diverse structural features of GLPs allow for the potential development of customized therapeutic approaches. Future research should focus on tailoring GLP structures to address specific medical challenges. For instance, optimizing the degree of branching and glycosidic linkages or engineering GLPs to maintain the triple helical structure could lead to tailored therapies with enhanced efficacy in the context of cancer treatment or immune modulation.

The various bioactive potentials of GLPs with different monosaccharide compositions open doors to precision medicine. Researchers can stratify patients based on their unique needs by understanding the specific structural attributes that lead to anti-inflammatory, antioxidant, anti-tumor, or immune-simulating effects. This individualized approach could lead to more effective and targeted treatments, reducing adverse effects and enhancing patient outcomes.

Exploring the synergy between GLPs and other therapeutic agents, such as existing cancer treatments, holds great promise. By combining GLPs with traditional pharmaceuticals, researchers may uncover novel synergistic effects that improve the overall treatment outcomes. This approach has the potential to revolutionize combination therapy in the field of oncology and beyond. Conducting long-term studies to assess the impact of GLPs on tumor progression, recurrence, and patient survival is of paramount importance. This research should examine the immediate effects of GLP-based treatments and their sustained benefits, offering a comprehensive perspective on their clinical utility for survivors and long-term patient management.

The safety and toxicity profiles of GLPs require rigorous evaluation before widespread clinical implementation. Comprehensive studies are necessary to ensure that GLPs are efficacious and safe for human consumption. Researchers can instill confidence in the medical community and patients by addressing these concerns.

Gaining a deeper understanding of the mechanisms underlying the interaction between GLP structures and immune receptors is pivotal. This knowledge can inform the development of more targeted therapies, focusing on precise interactions that enhance immune responses and suppress tumor growth. Investigating the role of PRRs, such as Dectin-1 and TLRs, will be central to these efforts.

## Anti-cancer properties of GLPs

**Fig.1:**
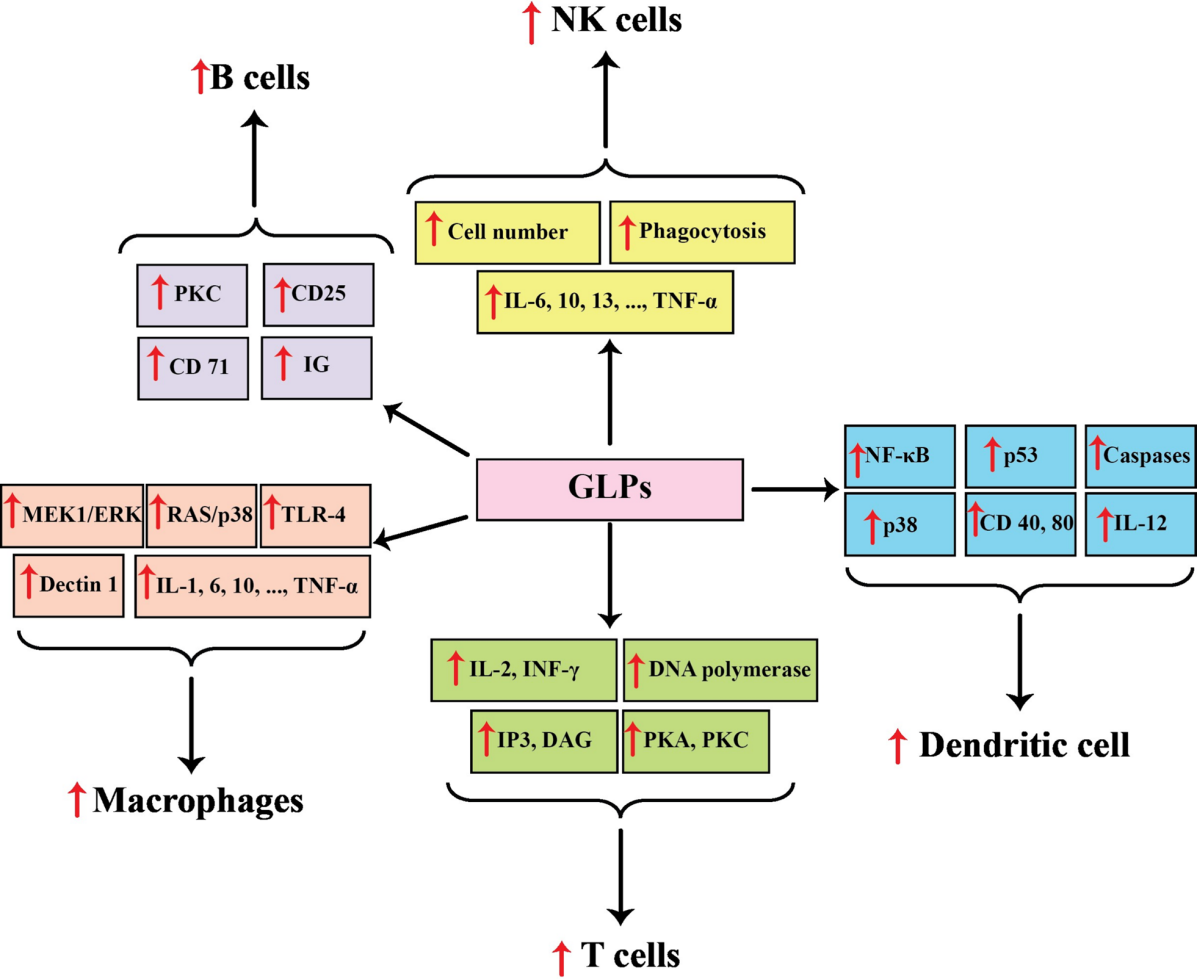
Immunomodulating effects of GLPs in different types of cancer

**Fig.2:**
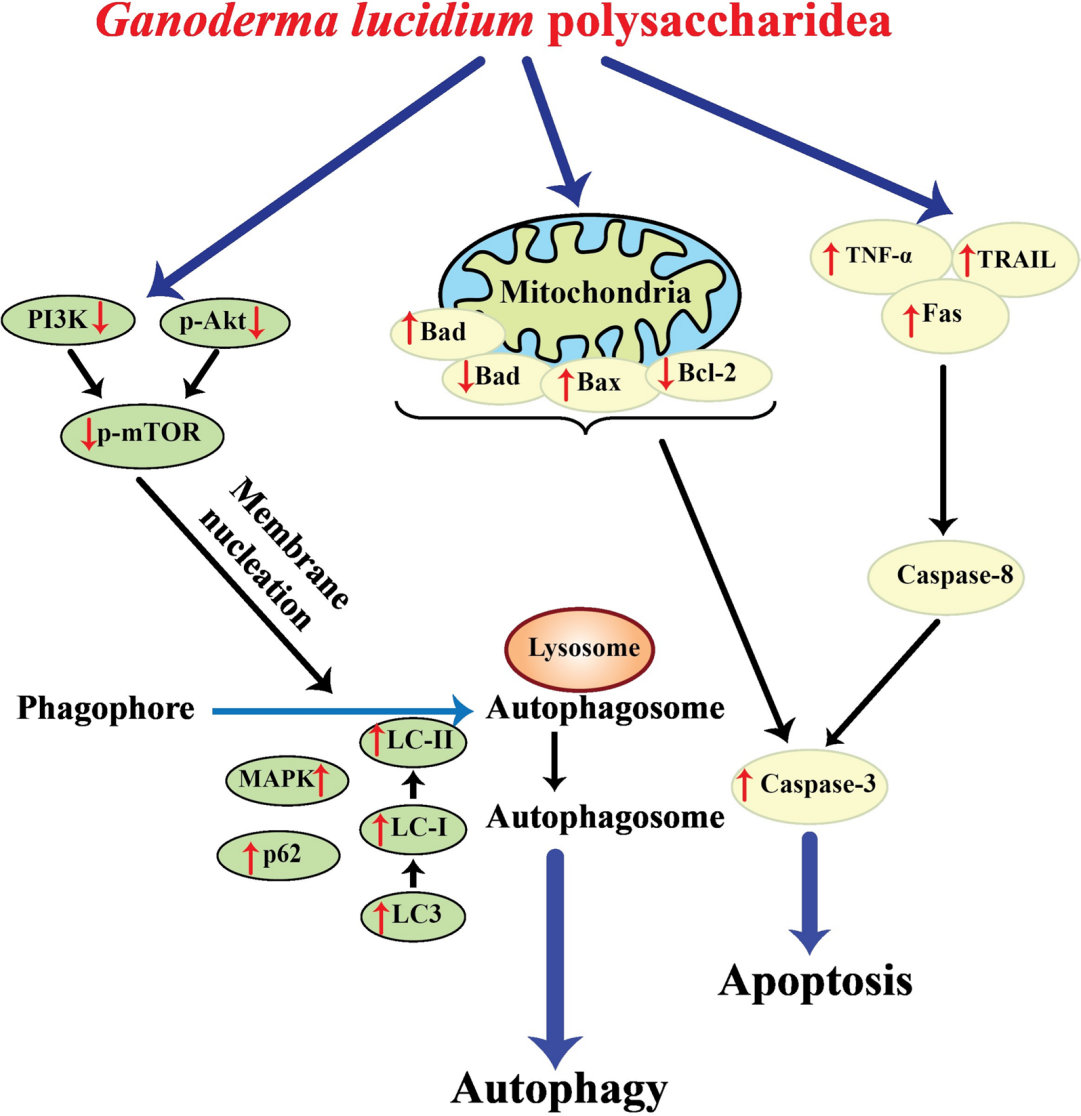
Mechanisms of cell death induction by GLPs

**Fig.3:**
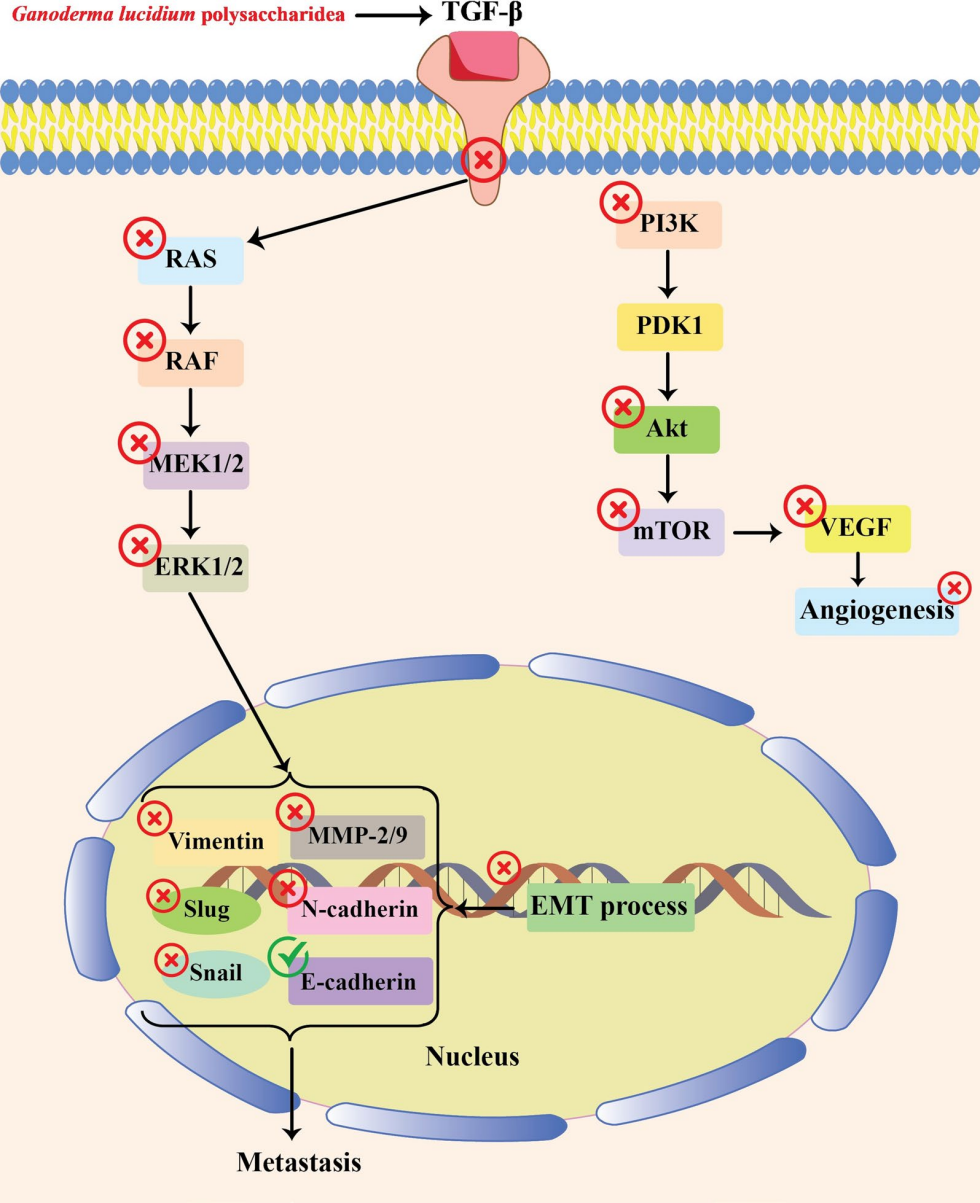
Molecular pathways associated with suppression of angiogenesis and metastasis by GLPs

Various studies have indicated that GLPs enjoy the capability of anti-tumor effects in different types of cancer (Table 2). Several mechanisms have been suggested for suppressing cancer development by GLPs. In this way, regulation of the immune system (Fig. 1), apoptosis induction (Fig. 2), and angiogenesis-metastasis inhibition (Fig. 3) are the main ones. The effects of GLPs on different cancers have been presented in the following sections.

**Table 2:** A brief of different studies related to the application of GLPs in cancer treatment

Cancer type	GLP dose	Study model	Effects	Mechanism	References
Breast	In vitro: 0–0.4 μM, 0–72 h	In vitro: MCF-7 cell lines	Decreased cell viability, arrested the cell cycle at the sub-G1 phase, induced apoptosis	↑: cytochrome C, activated caspase-3, -9, and PARP ↓: –	[95]
	In vivo: GLP: 200–400 mg/kg/day paclitaxel: 12.5 mg/kg, twice a week	In vivo: xenograft mice	Reduced tumor growth and size, restored the anti-cancer immune cells, and recovered gut microbiota dysbiosis stimulated by paclitaxel	↑: – ↓: GLUT3, LDHA, PDK	[109]
	In vitro: 0–160 μg/mL, 24 h In vivo: 8 mg/kg	In vitro: 4T1 cell lines In vivo: xenograft mice	Declined the number and size of 4T1 cells, increased radiosensitivity, reduced tumor growth, promoted apoptosis, and inhibited lung metastasis	↑: INF-γ/IL-4 ratio ↓:	[132]
	In vivo: Au-GLPs (30 mg/kg/every four days, 12 days), Dox (4 mg/kg)	In vivo: xenograft mice bearing 4T1 breast cancer tumors	Reduced tumor weight, decreased body weight loss rate, reduced pulmonary metastasis, and increased CD4 + and CD8 + T cell proliferation	↑: – ↓: –	[137]
Cervical	In vivo: 0–300 mg/kg/day, 40 days	In vivo: rats bearing cervical cancer	Increased antioxidant activity and reduced inflammation	↑: CAT, GSH-Px, and SOD ↓: IL-1β, IL-6, and TNF-α	[121]
	In vivo: GLPs (30 mg/kg/day, 14 days), cisplatin (5 mg/kg/day, 14 days)	In vivo: xenograft mice	Induced apoptosis, enhanced the spleen and thymus indexes, decreased the toxicity effects on hepatic and renal functions	↑: Bax ↓: Bcl-2	[144, 145]
	In vivo: 0–500 μg/mL, 0–72 h	In vitro: C-33A and HeLa cell lines	Reduced cell viability, induced apoptosis, arrested the cell cycle, suppressed the development of the EMT process	↑: Bax, cleaved caspase-3 and –9, E-cadherin, ↓: Bcl-2, N-cadherin, Slug, Vimentin, p- JAK, p-STAT5	[46]
Colon and Colorectal	In vitro: 0.625–5 mg/mL, 0–72 h	In vitro: HCT-116 cell lines	Reduced cell viability suppressed cell migration, changed cell morphology	↑: Ca^2+^, caspase-8, Fas ↓: -	[63, 64]
	In vitro: 0–10 mg/mL, 0–72 h	In vitro: HCT-116 cell lines	Reduced cell viability, arrested the cell cycle at the S phase, promoted apoptosis, and DNA fragmentation	↑: Bax to Bcl-2 ratios, caspase-3, caspase-9, PARP ↓:	[63, 64]
	In vitro: 0–10 mg/mL, 0–72 h	In vitro: LoVo cell lines	Declined cell viability, suppressed cell migration, induced apoptosis and DNA fragmentation	↑: Caspase-3, Caspase-8, Caspase-9, Fas, PARP ↓:	[65]
	In vitro: 200 μg/mL, 24 h	In vitro: HT29 (p53R273H) and SW480 (p53 R273H&P309S)	Promoted apoptosis, recovered p53	↑: Bax, p21, p53 ↓:	[44]
	In vitro: 0–7.5 mg/mL, 0–48 h In vivo: 0–300 mg/kg/day, six weeks	In vitro: HCT116 cell lines In vivo: Xenograft mice	Reduced cell viability, inhibited the cell cycle progression, stimulated apoptosis, decreased tumor growth	↑: caspase-3, caspase-9, NAG-1, p21 ↓: Bcl-2, cyclin A2, cyclin B1, Ki67, PCNA, survivin	[77]
	In vivo: 393.75 g/kg/day	In vivo: xenograft mice	Inhibited colon shortening, decreasing the mortality rate, declined the abundance of cecal Oscillospira, associated genes	↑: ↓: Scd1, Fabp4, Mgll, Acaa1b	[72]
	In vitro: 0–10 mg/mL, 0–72 h In vivo: 0–300 mg/kg/day, 14 days	In vitro: HT-29 and HCT-116 cell lines In vivo: xenograft mice	Reduced cell viability, induced autophagy, decreased tumor growth, and volume	↑: LC3-II, GFP-LC3 puncta ↓: -	[85]
	In vitro: 0–0.32 mg/mL, 24 h In vivo: 0–300 mg/kg/day, 14 days	In vitro: HT-29 cell lines In vivo: xenograft mice	Reduced inflammation	↑: ↓: COX-2, IL-1β, IL-6, iNOS, TNF-α, JNK, ERK	[33]
	In vitro: GLPs: 3 μg/mL, 72 h Paclitaxel: 0.5 μM, 72 h In vivo: 2 mg/kg/day, 30 days	In vitro: CT26 and HCT-15 cell lines In vivo: xenograft mice	Reduced cell growth and viability, inhibited tumor growth, and triggered apoptosis	↑: α-catenin, p53 ↓: IL-1β, IL-11, Cox-2	[66, 69]
Gastric	In vivo: 400 and 800 mg/kg/every two days, four weeks	In vivo: Wistar rats bearing gastric cancer	Reduced inflammation and increased antioxidant activity	↑: IL-2, IL-4, IL-6, CAT, GSH-Px, SOD ↓: IL-6 and TNF-α	[84]
	In vitro: 0–15 mg/mL	In vitro: AGS cell lines	Reduced cell viability, promoted apoptosis and autophagy	↑: cleaved-PARP, LC3-II, p62 ↓: Bcl-2, pro-caspase-3	[143]
Glioma	In vivo: 0–200 mg/kg/day, two weeks	In vivo: male Fischer rats bearing glioma	Reduces tumor size, and modulated host immune responses	↑: IL-2, TNF-α, INF-γ ↓: -	[113]
Hepatocellular	In vivo: 0–200 mg/kg/day, four weeks	In vivo: xenograft mice	Inhibited tumor growth	↑: IL-2, miR-125 ↓: FoxP3, Notch1	[56, 57]
Leukemia	In vitro: 0–200 μg/mL, 0–48 h	In vitro: THP-1 cell lines	Induced apoptosis degraded DNA	↑: TNF-α, caspase-3, caspase-7, TRAIL ↓: -	[16]
Lung	In vitro: 0- 12.8 μg/mL, 24 h	In vitro: lung cancer plasma patients	Reduced cell proliferation	↑: – ↓: –	[110]
	In vitro: 0–1000 μg/mL, 24 h In vivo: 2.5 g/kg/day, 14 days	In vitro: A549 cell lines In vivo: xenograft mice	Reduced the cell viability, and tumor weight, increased the immune index of serum	↑: – ↓: –	[34]
	In vitro: 0–300 μg/mL, 0–48 h In vivo: 75 mg/kg/every two days	In vitro: A549 cell lines In vivo: xenograft mice	Reduced the viability and mobility of lung cancer cells	↑: – ↓: EGF, p-Akt, p-ERK1/2, p-FAK, p-Smad2, TGF-β	[39]
	In vivo GLPs: 75 mg/kg/every two days, 20 days Cisplatin: 2.3 mg/kg/day, five days	In vivo: xenograft mice	Attenuated tumor growth and formation of nodular pulmonary metastases, induced apoptosis, and enhanced the therapeutic effects of cisplatin	↑: – ↓: –	[88]
	In vitro: 0–800 μg/mL, 0–72 h	In vitro: A549 and LLC1 cell lines	Alleviated the growth and viability of both cell lines	↑: - ↓: p-Akt, p-EGFR, p-ERK, p-Smad2, p-FAK, Slug, twist, TGFβRII	[89]
Oral	In vitro: 0–800 μg/mL, 0–72 h	In vitro: SAS cell lines	Declined the viability of cells, suppressed the cell cycle, prompted apoptotic responses, reduced cytotoxicity of cisplatin	↑: Bax/Bcl-2 ratio ↓: p-Akt, p-EGFR	[38]
	In vitro: 0.01- 15 mg/mL, 72 h	In vitro: SCC-9 cell lines	Reduced the viability and colony formation of SCC-9 cells, delayed cell migration, and inhibited EMT development	↑: - ↓: ABCG2, AXL, N-cadherin, p75NGFR Twist, Vimentin	[18]
Ovarian	In vivo: 100–300 mg/kg/ twice a day	In vivo: ovarian cancer rats	Reduced malondialdehyde formation and increased the total antioxidant capacity	↑: SOD, CAT, GSH ↓: -	[131]
	In vitro: 0–10 μg/mL, 0–3 days	In vitro: OVCAR-3 cell lines	Reduced cell growth and viability and inhibited the cell cycle	↑: CAT, SOD, NQO1, GSTP1, DJ1, Trx, Nrf-2 ↓: cyclin D1	[36]
Prostate	In vitro: 0–20 μg/mL, 0–120 h	In vitro: LNCaP cell lines	Reduced cell proliferation and migration,, suppressed the cell cycle at the G1 stage	↑: p21 ↓: PRMT6, CDK2, FAK, SRC	[138]
	In vitro: 1.25–10 mg/mL, 0–72 h	In vitro: PC-3 cell lines	Reduced the cell viability, stimulated late apoptosis	↑: NAG-1, cleaved PARP ↓: pro-caspase-3, -6, and -9, p-Akt, p-MAPK/ERK	[120]
Sarcoma	In vivo: 0–100 mg/kg/day, 21 days	In vivo: xenograft mice	Reduced the tumor growth and size	↑: – ↓: –	[25]
Skin	In vivo: 33.3–300 mg/kg/day, eight days	In vivo: xenograft mice	Inhibited tumor growth	↑:– ↓:–	[86]

### Breast cancer

In an investigation by Shang, Li et al. [95], Se-GLPs, selenium-containing polysaccharides, were isolated from Se-enriched *G.lucidum* for the first time. Treatment of MCF-7 breast cancer cells with Se-GLPs (0–0.4 μM, 0–72 h) decreased cell viability in a time- and dose-dependent fashion and arrested the cell cycle at the sub-G1 phase. Molecular analysis revealed that Se-GLPs disorganize the mitochondrial membrane potential, increasing the cytosolic content of cytochrome C, activated caspase-3, -9, and PARP. Thus, Se-GLPs promoted apoptosis in breast cancer cells through a mitochondria-dependent pathway and decreased cell proliferation. Compelling evidence has displayed the fundamental function of gut microbiota in carcinogenesis and chemotherapy results. Notably, paclitaxel, a primary chemotherapeutic agent for breast cancer, is associated with safety concerns and efficacy limitations due to severe adverse effects and drug resistance. Su, Li et al. [109] conducted a study involving xenograft mice with metastatic breast cancer to address this issue. They investigated the impact of GLPs (administered at 200–400 mg/kg/day) in combination with paclitaxel (administered at 12.5 mg/kg, twice a week). The results of this study indicated that the combined therapy effectively reduced tumor growth and size. Furthermore, the treatment led to the downregulation of Warburg effect-associated proteins, including glucose transporter 3 (GLUT3), lactate dehydrogenase A (LDHA), and pyruvate dehydrogenase kinase (PDK), resulting in the suppression of glucose uptake. Metabolic changes were observed in tumor metabolites, with increased hydroxylamine and decanoyl carnitine, alongside a reduction in 3-hydroxypyridine, 6-phosphonoglucono-D-lactone, gluconolactone, L-sorbose, and L-alpha-aminobutyric acid.

Additionally, the combination of paclitaxel and GLPs exhibited the capacity to rejuvenate exhausted anti-cancer immune cells by suppressing the regulation of immune checkpoints, including PD-1 and Tim3. In contrast, paclitaxel administered alone increased the level of CTLA-4. The study on gut microbiota also revealed that the co-treatment with paclitaxel and GLPs reversed the gut microbiota dysbiosis induced by paclitaxel, including an increase in *Bacteroides* and *Ruminococcus* and a decrease in cancer risk genera, *Desulfovibrio* and *Odoribacter*. The study also found a negative correlation between the elevated levels of *Ruminococcus* and the content of fructose-6-phosphate in tumor cells. These findings suggest that the combination therapeutic strategy holds promise against breast cancer, potentially attributed to the modulation of tumor metabolism and gut microbiota. The study also found a negative correlation between the elevated levels of *Ruminococcus* and the content of fructose-6-phosphate in tumor cells. These findings suggest that the combination therapeutic strategy holds promise against breast cancer, potentially attributed to the modulation of tumor metabolism and gut microbiota.

### Cervical cancer

#### Impact of GLPs on cervical cancer: an in vivo setting

Cervix carcinoma stands as a significant global health concern, ranking as the second most common cancer among females worldwide. Particularly in developing countries, it presents a formidable challenge, being the leading cause of cancer-related mortality. To address this pressing issue, a study by XiaoPing, Yan et al. [121] investigated the potential of GLPs in the context of cervical cancer. In a rat model of cervical cancer, GLPs were administered at varying doses (ranging from 0 to 300 mg/kg/day over 40 days). The study revealed that GLPs exhibited a dose-dependent effect, resulting in increased levels of antioxidant enzymes, including catalase (CAT), glutathione peroxidase (GSH-Px), and superoxide dismutase (SOD). Moreover, GLPs reduced the expression of pro-inflammatory cytokines, such as IL-1β (interleukin-1β), IL-6 (interleukin-6), and TNF-α (tumor necrosis factor-alpha), in rats with cervical cancer. These findings suggest that GLPs may hold promise in enhancing antioxidant activity and modulating immune responses in cervical cancer.

#### GLPs as adjunct to cisplatin in cervical carcinoma

Cisplatin has long been recognized as a primary therapeutic agent for cervical carcinoma, although severe adverse effects, including nephrotoxicity and neurotoxicity, limit its use. In response to this limitation, a study sought to investigate the potential of GLPs as a complementary therapy to cisplatin in xenograft mice bearing cervical cancer. The co-treatment involved GLPs administered at 30 mg/kg/day and cisplatin at 5 mg/kg/day for 14 days. Interestingly, the results showed that this combination did not suppress tumor growth but yielded significant benefits. It enhanced spleen and thymus indexes and mitigated the toxic effects on hepatic and renal functions, thus improving the overall health of the subjects. Mechanistic insights revealed that GLPs exert their beneficial effects by inducing apoptosis, as evidenced by the upregulation of pro-apoptotic factor Bax and the downregulation of anti-apoptotic factor Bcl-2 Zhu, Xu et al. [144, 145].

#### Effect of GLP on cervical cancer cells: an in vitro approach

Building on the potential of GLPs in cervical cancer, a study by Jin, Song et al. [46], delved into the direct effects of GLPs on cervical cancer cells. The research investigated two cervical cancer cell lines, C-33A and HeLa, treating them with GLPs at varying concentrations (ranging from 0 to 500 μg/mL) over different time frames (up to 72 h). The results were compelling, demonstrating that GLPs exerted a dose- and time-dependent reduction in cell viability for both C-33A and HeLa cells. Furthermore, GLPs induced apoptosis in these cells, marked by increased pro-apoptotic proteins, including Bax and cleaved caspase-3 and -9, and decreased anti-apoptotic factor Bcl-2. Cell cycle analysis indicated that GLPs led to cell cycle arrest, primarily at the G0/G1 phase for C-33A cells and both the G0/G1 and S phases for HeLa cells. Additionally, GLPs effectively suppressed the epithelial-mesenchymal transition (EMT) process in cervical cancer cells, as evidenced by increased expression of E-cadherin and downregulation of N-cadherin, Slug, and Vimentin. The study also revealed that GLPs significantly reduced the phosphorylation of JAK and STAT5, indicating their impact on the EMT and JAK/STAT5 signaling pathways, ultimately inhibiting the viability and growth of cancer cells.

### Effect of GLP on colon and colorectal cancer

#### Impacts of GLPs on cell cycle and apoptosis

Colorectal cancer, a malignancy responsible for approximately 500,000 annual deaths and 940,000 new cases, remains a formidable global health concern. Liang, Guo et al. [63, 64] conducted a study to examine the potential of GLPs in the context of colorectal cancer. Their investigation involved treating HCT-116 colon cancer cells with GLPs at various concentrations (0.625–5 mg/mL) over 72 h. The results were striking, as GLPs significantly reduced cell viability. Notably, this reduction was linked to a cascade of effects, including cell migration suppression, cell morphology alterations, increased lactate dehydrogenase (LDH) release, and augmented intracellular calcium levels.

Furthermore, GLPs were found to enhance the expression of caspase-8 and Fas, which are key components of the death receptor pathway. Thus, GLPs were shown to inhibit tumorigenesis development in HCT-116 colon cancer cells, primarily by stimulating intracellular calcium release and the death receptor pathway. The anti-cancer potential of GLPs was further explored in the context of colorectal cancer cells by Liang, Yi et al. [63, 64]. In this study, HCT-116 cells were treated with GLPs at varying doses (0–10 mg/mL) over a 72-h period. The findings demonstrated that GLPs exerted a time- and dose-dependent reduction in cell viability. Beyond that, GLPs induced cell cycle arrest, notably at the S phase, and promoted cell apoptosis. This apoptotic process was characterized by the activation of caspase-3 and -9, DNA fragmentation, changes in cell morphology, and mitochondrial membrane depolarization. The ratio of Bax to Bcl-2 increased, while caspase-3, caspase-9, and poly(ADP-ribose) polymerase (PARP) were overexpressed. Intriguingly, the suppression of c-Jun N-terminal kinase (JNK) by SP00125 dramatically reduced the apoptosis promoted by GLPs. These results unveiled the capacity of GLPs to induce apoptosis in HTC-166 colon cancer cells, primarily through the overexpression of JNK via the mitogen-activated protein kinase (MAPK) pathway.

GLPs displayed their anti-cancer prowess not only in HCT-116 cells but also in LoVo colon cancer cell lines. In a study by Liang, Yi et al. [65], GLPs treatment (0–10 mg/mL, 0–72 h) reduced cell viability, inhibited cell migration, and induced DNA fragmentation, morphological changes, and LDH release. The mechanism underlying these effects involved the activation of caspase-3, -8, and -9, the overexpression of caspase-3 and Fas proteins, and the reduction of cleaved PARP. These findings emphasized the role of the Fas-caspase-mediated apoptosis pathway in the anti-cancer function of GLPs, alongside their capacity to inhibit cell migration. BSGLPs (sporoderm-broken spores of GLPs) were examined for their impact on colorectal cancer cells. In an incubation study involving HCT116 cells, BSGLPs (0–7.5 mg/mL, 0–48 h) demonstrated significant reductions in cell viability in a dose- and time-dependent manner. Additionally, BSGLPs influenced the cell cycle progression by downregulating cyclin A2 and cyclin B1 and upregulating p21. Apoptosis in cancer cells was stimulated through the downregulation of Bcl-2 and survivin at the mRNA level, as well as modulations of Bcl-2, PARP, pro-caspase-3, and -9 at the protein level. In vivo studies with xenograft mice further illustrated that BSGLPs (0–300 mg/kg/day, six weeks) could suppress the carcinogenesis of colorectal cancer cells by modulating genes related to cell cycle, proliferation, and apoptosis [77]. The interactions between GLPs and p53 in the context of colorectal cancer were explored in a separate study. It was noted that GLPs interacted with mutant p53, a prevalent feature in many cancers. GLPs treatment (200 μg/mL, 24 h), either alone or in conjunction with 5-fluorouracil, led to the restoration of mutant p53 in colorectal cancer cells (HT29 (p53^R273H^) and SW480 (p53 ^R273H&P309S^)). This restoration further promoted apoptosis, evidenced by overexpression of Bax and p21, and cell death. The recovery of p53 affected both transcriptional-dependent and independent axes, underscoring its crucial role in facilitating the anti-cancer functions of GLPs [44].

#### Autophagy-inducing and anti-inflammatory effects of GLPs

GLPs (0–10 mg/mL, 0–72 h) were found to induce autophagy in HT-29 and HCT-116 colorectal cancer cells, characterized by the upregulation of markers such as LC3-II, GFP-LC3 puncta, and double-membrane vacuoles. This induction of autophagy was linked to apoptosis induction, mediated by MAPK/ERK activation. Finally, in vivo experiments involving xenograft male BALB/C nude mice further corroborated the potential of GLPs in reducing tumor growth and increasing survival rates [83]. The anti-inflammatory potential of GLPs in colorectal cancer was explored in HT-29 cells. GLPs (0–0.32 mg/mL, 24 h) were found to downregulate pro-inflammatory markers such as COX-2, IL-1β, IL-6, iNOS, and TNF-α. Furthermore, the modulation of the inflammatory signaling axis, including JNK and ERK, was significantly reduced in xenograft male C57BL/6 mice treated with GLPs mice (0–300 mg/kg/day, 14 days). This finding suggested that GLPs may exert their anti-cancer and anti-inflammatory potential by inactivating the MAPK/JNK and ERK signaling pathways [33].

#### Chemo-Sensitization effects of GLPs

In a separate study, the combination of paclitaxel (0. 5 μM, 72 h) and GLPs (3 μg/mL, 72 h) showed promise as a chemo-sensitizing strategy in CT26 and HCT-15 colorectal cancer cells. While paclitaxel alone had a modest inhibitory effect on certain cell lines, the co-treatment with GLPs significantly reduced cell growth and viability. This combined therapy was effective in inhibiting tumor growth and triggering apoptosis. RNA-seq analysis revealed that GLPs decreased the regulation of the NF-κB-modulated inflammatory axis and overexpressed α-catenin and p53 tumor suppressors, providing a comprehensive understanding of their chemo-sensitizing effects. These findings collectively highlight the multifaceted potential of GLPs in combating colorectal cancer through various mechanisms, including apoptosis induction, cell cycle regulation, modulation of gut microbiota, and anti-inflammatory activities [66, 69].

### Effect of GLP on Gastric cancer

In an effort to evaluate the effects of GLPs in the context of gastric cancer, a study conducted by Pan, Jiang et al. [84] utilized Wistar rats bearing gastric cancer induced by MNNG (Methylnitronitrosoguanidine). The treatment involved the administration of GLPs at varying doses (400 and 800 mg/kg every two days) over four weeks. The results revealed a significant increase in the levels of specific cytokines, namely IL-2 (interleukin-2), IL-4 (interleukin-4), and IL-6, in a dose-dependent manner. IL-2, generated by activated T cells, plays a pivotal role in stimulating immunoregulatory effects on various immune cells [104]. IL-4, on the other hand, is involved in the formation of IgE through the differentiation of pre-T helper cells into Th2 cells, leading to the switch in the immunoglobulin class from IgM/IgG to IgE in B cells. IL-4 also inhibits the synthesis of INF-γ (interferon-gamma) and influences Th1 cell differentiation [87]. Moreover, IL-10 (interleukin-10), a crucial immunomodulatory cytokine, contributes to the modulation of inflammatory responses, primarily through its impact on TNF-α production [43]. The inflammation is a consequence of the over-expression of pro-inflammatory cytokines such as IL-6 and TNF-α and has a crucial function in synovial inflammation [93]. Notably, the initiation of MNNG in rats led to the activation of inflammatory cells and the release of pro-inflammatory cytokines, such as IL-6 and TNF-α. However, treatment of gastric cancer-bearing rats with GLPs effectively reduced the levels of IL-6 and TNF-α. An increase in the levels of CAT, GSH-Px, and SOD accompanied this anti-inflammatory response. In contrast, MNNG-induced oxidative stress led to a decrease in the levels of these antioxidant enzymes. These findings underscored the ability of GLPs to counteract the inflammation and oxidative stress associated with gastric cancer [84].

In another investigation involving gastric cancer, Zhong, Fang et al. [143] conducted an in vitro study by incubating gastric cancer cells (AGS) with sporoderm-removed spores GLP at various concentrations (0–15 mg/mL). The results of this study demonstrated that GLPs significantly reduced cell viability and promoted apoptosis. These effects were accompanied by a reduction in the expression of the anti-apoptotic protein Bcl-2 and pro-caspase-3, along with an increase in cleaved-PARP, indicating apoptosis induction. Furthermore, GLPs were found to stimulate autophagy, as evidenced by the modulation of LC3-II and p62. However, the autophagy process exhibited some disruption, particularly in autophagy flux within cancer cells, leading to the accumulation of autophagosomes. These findings provide insights into the potential of GLPs, specifically sporoderm-removed spores GLP, as a regulator of autophagy in gastric and potentially other types of cancer. Overall, the studies discussed in this section shed light on the multifaceted impact of GLPs in gastric cancer, involving the modulation of cytokines, anti-inflammatory responses, and the regulation of processes like apoptosis and autophagy.

### Interactions between GLPs and Glioma cancer

Glioma, a highly recurrent and lethal brain cancer, has been the focus of a study conducted by Wang, Shi et al. [113]. In this in vivo experiment, male Fischer rats afflicted with glioma were subjected to treatment with GLPs at varying doses (ranging from 0 to 200 mg/kg/day) over a two-week period. The results of this study revealed several significant findings regarding the potential of GLPs in the context of glioma. Administering GLPs led to an augmentation in the serum levels of crucial cytokines, including IL-2, TNF-α, and INF-γ. These cytokines are known for their pivotal roles in modulating immune responses, particularly in bolstering the ability of the body to combat cancer. Additionally, GLPs were found to enhance the cytotoxic effects of both NK and T cells, essential components of the immune system responsible for detecting and eliminating cancer cells. Moreover, GLPs contributed to the functional maturation of dendritic cells, a class of immune cells responsible for presenting antigens to T cells, thereby enhancing the adaptive immune response. The impact of GLPs on glioma was profound, as the treatment effectively inhibited the growth of glioma tumors, leading to a reduction in tumor size. This tumor-suppressive effect translated into an increase in the survival rates of the rats afflicted with glioma. These findings collectively point to the anti-cancer potential of GLPs, particularly in the context of glioma, through their remarkable ability to modulate and enhance host immune responses. In summary, the study by Wang, Shi et al. [113] underscores the promising role of GLPs in glioma therapy, emphasizing their capacity to bolster immune responses, inhibit tumor growth, and improve the survival rates of glioma-afflicted individuals.

### Imacts of GLPs and Hepatocellular carcinoma

Hepatocellular carcinoma, one of the most lethal cancer types globally, presents significant challenges regarding chemotherapy efficacy. In a critical study by Li, Shuai et al. [56], the potential of GLPs in combating hepatocellular carcinoma was explored. The study utilized hepatocellular carcinoma-bearing xenograft mice and administered GLPs at varying doses (ranging from 0 to 200 mg/kg every two days) over a span of four weeks. The results of this study revealed the profound impact of GLPs on inhibiting tumor growth. This effect was closely linked to the ratio alteration between two critical types of T cells, specifically effector T cells (Teff) and regulatory T cells (Treg). Treg cells are known to exert an immunosuppressive influence on Teff cells, and their balance is crucial in immune regulation.

Mechanistically, GLPs were found to suppress the Treg-associated inhibition of Teff cell proliferation by elevating the secretion of IL-2. IL-2 is a critical cytokine in immune responses, playing a significant role in the activation and proliferation of various immune cells, including Teff cells. Furthermore, treating hepatoma-bearing mice with GLPs had a noteworthy impact on the intratumoral Treg cells. GLPs were observed to elevate significantly the levels of miR-125 within these Treg cells. Subsequent investigations delved into the specific molecular mechanisms involved. It was revealed that GLPs, through the upregulation of miR-125b, reduced FoxP3 expression in Treg cells. FoxP3 is a crucial transcription factor associated with Treg cells and is known for its role in maintaining their immunosuppressive functions. Notably, the reduction of FoxP3 expression was linked to a decrease in the expression of Notch1, a key regulator in cellular signaling pathways.

These findings collectively highlight the potential of GLPs in hepatocellular carcinoma therapy. By altering the balance between Teff and Treg cells, modulating IL-2 secretion, and regulating miR-125b and FoxP3 expression, GLPs demonstrate a multifaceted impact on the immune response against hepatocellular carcinoma.

### Effects of GLPs on Leukemia

In the context of the immunoregulating and anti-cancer effects of GLPs, Cheng, Huang et al. [16] investigated the anti-tumor activity of GLPs in human leukemia THP-1 cells. Their research revealed that GLPs notably impacted inducing apoptosis in THP-1 cells, primarily through the activation of death receptors DR3 and DR4/5. Specifically, when THP-1 cells were exposed to varying concentrations of GLPs (ranging from 0 to 200 μg/mL) for different time intervals (up to 48 h), several significant observations were made: GLPs induced a dose-dependent increase in the expression of TNF-α. The treated cells exhibited alterations in their cellular morphology. DNA within the cells was degraded. Cells displayed a tendency to aggregate. Apoptosis, a controlled form of cell death, was triggered. The mechanism behind these effects involved GLPs stimulating TNF-α and TRAIL (TNF-related apoptosis-inducing ligand), which, in turn, initiated signaling through the oligomerization of death receptors. This signaling cascade led to the activation of caspase-3 and -7, crucial enzymes responsible for executing the process of apoptosis. In summary, GLPs were found to induce apoptosis in THP-1 cells by activating specific adaptor proteins and the caspase pathway through the TNF-α and TRAIL pathways. This study highlights the potential of GLPs in exerting anti-tumor effects by promoting programmed cell death in leukemia cells.

### GLPs in lung cancer management

Lung cancer stands as one of the leading global causes of mortality. A compelling study conducted by Sun, Li et al. [110] delved into the potential of GLPs to counteract plasma-induced suppression of lymphocytes in lung cancer patients. Lymphocytes, integral to the immune response against cancer, displayed suppressed proliferation, CD69 modulation, and reduced perforin and granzyme B formation when exposed to plasma from lung cancer patients after phytohemagglutinin (PHA) activation. However, incubation of lymphocytes from lung cancer patients with GLPs (ranging from 0 to 12.8 μg/mL over 24 h) effectively reversed these immunosuppressive changes. This intriguing outcome suggests that GLPs may hold promise as an adjunctive treatment in cancer management by mitigating the immune suppression frequently encountered in lung cancer patients. Another significant contribution by Hsu, Qiu et al. [39] further elucidated the potential of GLPs in lung cancer treatment. Using A549 cell lines, the study demonstrated that GLPs, over 48 h within a concentration range of 0 to 300 μg/mL, significantly reduced the viability and mobility of lung cancer cells. At the molecular level, GLPs exerted their anti-cancer effects by decreasing the phosphorylation levels of Akt, ERK1/2, FAK, and Smad2. Importantly, they induced the degradation of receptors for epidermal growth factor (EGF) and transforming growth factor-beta (TGF-β) within lysosomes and proteasomes, respectively. In vivo experiments provided compelling evidence of the anti-tumor activities of GLPs, showcasing their ability to inhibit lung tumor growth, reduce the volume of metastatic nodules in the lung, and improve the survival rate of lung cancer-bearing mice.

In a remarkable revelation by Qiu, Hsu et al. [88], the co-treatment of xenograft mice bearing lung cancer with GLPs and cisplatin exhibited a synergistic suppressive effect on tumor growth, nodular pulmonary metastases and promoted apoptosis. This finding highlights the potential of GLPs as a supplementary or adjuvant compound in cancer treatment, notably enhancing the therapeutic effects of cisplatin.

Further expanding the scope, Fang, Zhao et al. [22] explored the anti-cancer properties of polysaccharides extracted from sporoderm-removed spores of *G. lucidum* (RSGLP) and sporoderm-broken spores (BSGLP). Their research encompassed various cancer cell lines, including breast, colon, liver, and lung. The study revealed that RSGLP exhibited more potent inhibitory effects on cell viability and apoptosis induction in comparison to BSGLP. Additionally, RSGLP displayed greater efficacy in reducing tumor growth in HCT116 and NCI-H460 xenograft mice and suppressing tumor-induced splenomegaly in nude mice. RSGLP also demonstrated superior anti-inflammatory activities by significantly reducing serum inflammatory cytokine levels and inhibiting the activation of macrophage RAW264.7, along with the modulation of key inflammatory mediators, such as COX-2, IL-1β, iNOS, and TNF-α. This marked difference in performance highlights the potential of RSGLP as a novel anti-cancer agent, offering both anti-tumor and immune-stimulating effects.

In a parallel investigation by Qiu, Lo et al. [89], lung carcinoma cell lines (A549 and LLC1) were exposed to GLPs over 72 h at concentrations ranging from 0 to 800 μg/mL. The outcomes were impressive, with GLPs effectively suppressing the growth and viability of both cell lines. At the molecular level, they exerted these effects by attenuating the phosphorylation of Akt, EGFR, and ERK, downregulating TGFβRII, and reducing the phosphorylation of downstream molecules such as Smad2 and FAK. Moreover, the regulation of transcription factors Slug and twist, involved in drug resistance and cell survival, was decreased.

These studies collectively demonstrate the potential of GLPs in managing lung cancer, both by enhancing immune responses and directly inhibiting cancer cell growth and metastasis. The various facets of their activity indicate their promise as a versatile tool in the fight against this deadly disease.

### Oral cancer and the promising role of GLPs

Tongue cancer, a type of oral cancer, is particularly prevalent in Southeast Asia due to dietary habits, and the development of targeted therapeutic agents for effectively suppressing this cancer has been a significant challenge. In a study conducted by Hsu, Hua et al. [38], the anti-cancer activities of GLPs were explored in the context of human tongue cancer. The findings revealed that GLPs, administered at various concentrations over a 72-h period, substantially reduced the colony formation and viability of SAS tongue cancer cells. Notably, GLPs induced cell cycle arrest at the subG1 and G2/M phases, facilitated apoptotic responses and increased the Bax/Bcl-2 ratio associated with apoptosis. Mechanistically, GLPs were found to inhibit the phosphorylation of Akt and EGFR, contributing to their role in suppressing cancer cell growth. Additionally, GLPs exhibited a remarkable capacity to decrease the inhibition concentration (IC50) of cisplatin. When combined, GLPs and cisplatin showed a synergistic interaction, resulting in reduced cell viability and enhanced apoptosis induction. Importantly, GLPs were able to mitigate the cisplatin-induced cytotoxicity in normal human tongue SG cells.

Oral squamous cell carcinoma (OSCC), a form of oral cancer, is witnessing an increasing incidence globally, posing a significant health concern. Consequently, a study aimed to evaluate the impact of GLPs (administered at varying concentrations over 72 h) on OSCC, particularly on SCC-9 cells. GLPs treatment effectively reduced the viability and colony formation of SCC-9 cells, hindered cell migration, and caused alterations in cell morphology characterized by increased size and granularity and EMT development. Further investigations unveiled that GLPs were responsible for decreasing the expression of several crucial markers associated with EMT (AXL, N-cadherin, Twist, Vimentin), a cancer stem cell (CSC) marker (p75NGFR), and a type of ATP-binding cassette (ABC) transporter (ABCG2). These results underscore the potential of GLPs as a promising therapeutic agent for managing OSCC [18]

The increasing prevalence of tongue cancer and OSCC worldwide highlights the pressing need for effective treatments, and GLPs offer a potential avenue for addressing these challenges through their demonstrated anti-cancer properties. Their capacity to induce apoptosis, inhibit cell cycle progression, and enhance the efficacy of existing therapeutic agents like cisplatin, while sparing normal cells, positions GLPs as valuable candidates in the battle against oral cancers.

### Ovarian cancer and GLPs as a therapeutic agent

#### Ovarian Cancer and the Antioxidant Effects of GLPs

Ovarian cancer ranks among the prominent causes of cancer-related deaths in women. YouGuo, ZongJi et al. [131], delved into the effects of GLPs on serum antioxidant enzymes in ovarian cancer rats. Through their study, they observed that administering GLPs (100–300 mg/kg, twice daily) to ovarian cancer rats led to a decrease in malondialdehyde formation and an increase in the activity of SOD, CAT, GSH-Px, and the total antioxidant capacity. This outcome strongly suggested the antioxidant properties of GLPs, emphasizing their potential advantages in treating ovarian cancer.

#### *G. lucidum* extract and ovarian cancer cells

Hsieh and Wu [36] offered insights into applying *G. lucidum* extract (GLE) in the context of ovarian cancer. Their study focused on the effects of GLE on OVCAR-3 cells. GLE treatment at varying concentrations (0–10 μg/mL) for different durations (0–3 days) resulted in a dose- and time-dependent reduction in cell growth and viability. The impact of this treatment was further reflected in cell cycle inhibition, facilitated by the downregulation of cyclin D1. Importantly, GLE exhibited chemoprotective activities against cancer, which were associated with promoting antioxidant enzymes, namely CAT and SOD, as well as phase II detoxification enzymes (NQO1 and GSTP1). Subsequent investigations elucidated that GLE achieved these effects by augmenting the activities of these enzymes through the upregulation of DJ1 and Trx. Both DJ1 and Trx play pivotal roles in stabilizing and enhancing the functional properties of Nrf-2, a key player in the cellular antioxidant response. Consequently, GLE was found to induce the Nrf-2-associated activation of the antioxidant response element (ARE) by boosting nuclear levels of Nrf-2, enhancing its stability, and fortifying its capacity to interact with ARE-responsive genes. These findings shed light on the potential of GLE as a valuable therapeutic agent, particularly in its role in modulating the antioxidant response against ovarian cancer [36]

The prevalence and impact of ovarian cancer make developing novel therapeutic strategies crucial, and the antioxidant properties of GLPs hold promise in this context. Additionally, the observed chemoprotective effects of GLE in ovarian cancer cells, specifically its role in promoting antioxidant enzymes and detoxification pathways, offer a potential avenue for future research and therapeutic development in the fight against this devastating disease.

#### Effect of GLPs on Prostate cancer

Prostate cancer represents one of the most pervasive malignancies affecting men, with a concerning increase in both its incidence and mortality rates. In a study by Zhao, Zhou et al. [140], the potential of GLPs in combating prostate cancer was explored. The research involved the treatment of LNCaP prostate cancer cells with GLPs over a range of concentrations (0–20 μg/mL) and durations (0–120 h). The outcomes demonstrated a significant reduction in cell proliferation and migration, with a concurrent cell cycle arrest at the G1 stage. On the molecular level, the study revealed that GLPs exerted their effects by downregulating the expression of essential proteins such as PRMT6, CDK2, FAK, and SRC, while simultaneously upregulating the expression of p21 in LNCaP cells. The modulation of the PRMT6 signaling pathway emerged as a central mechanism through which GLPs functioned as tumor inhibitors in prostate cancer cells.

NAG-1, known for its proapoptotic activities, is promising in cancer prevention and treatment. In a study by Wu, Na et al. [120], GLPs were investigated for their potential to mitigate prostate cancer, specifically focusing on PC-3 prostate cancer cells. GLPs were administered over various concentrations (1.25–10 mg/mL) and timeframes (0–72 h), leading to a dose- and time-dependent reduction in the viability of PC-3 cells. This treatment induced late-stage apoptosis, as evident from PARP cleavage and the suppression of pro-caspase-3, -6, and -9 expression. Notably, GLPs demonstrated an ability to upregulate the expression of NAG-1 and its transcriptional factor, early growth response-1, in a dose- and time-dependent manner. The subsequent investigations unveiled that GLPs significantly increased the promoter activity of NAG-1, suggesting that GLPs may exert their influence on NAG-1 through transcriptional regulation. Moreover, GLPs were found to suppress the phosphorylation of Akt and MAPK/ERK in PC-3 cells. This dual mode of action was instrumental in controlling prostate cancer by stimulating apoptosis while concurrently blocking the Akt and MAPK/ERK signaling pathways.

The multifaceted approach exhibited by GLPs, involving the inhibition of PRMT6 signaling and the activation of NAG-1, combined with the regulation of Akt and MAPK/ERK pathways, underscores their potential as a valuable agent in combating prostate cancer. These findings shed light on promising avenues for therapeutic interventions and exploring GLProle in prostate cancer treatment.

#### Impact of GLPs on Sarcoma

Fu, Shi et al. [25] conducted a study in which they extracted water-soluble GLPs from *G. lucidum* and assessed their potential as anti-tumor agents. The research involved the treatment of xenograft mice bearing sarcoma (S180 cells) with these water-soluble GLPs, administered at various concentrations (0–100 mg/kg/day) over a span of 21 days. The results demonstrated a significant and dose-dependent reduction in tumor growth and size, highlighting the anti-tumor efficacy of these water-soluble GLPs.

### Interaction between GLPs and skin cancer cells

Administration of GLPs (33.3–300 mg/kg/day, eight days) in xenograft mice bearing EAC tumors inhibited tumor growth significantly in a dose-dependent manner. GLPs also elevated phagocytic coefficient, phagocytic index, and 50% hemolysin value, elucidating the potential immunomodulating effects of GLPs [86].

In this section, the multifaceted potential of GLPs in the context of various cancer types has been explored. Our analysis of the existing literature reveals compelling evidence of the anti-cancer properties of GLPs, which are attributed to their ability to modulate key pathways involved in cancer development and progression. Across different cancer types, GLPs have demonstrated the capacity to inhibit cell growth, induce apoptosis, and suppress the migration and invasion of cancer cells. These effects were often mediated by regulating essential proteins involved in apoptosis, cell cycle control, and EMT. Moreover, the immunomodulatory properties of GLPs have been highlighted in various studies, where they rejuvenated exhausted anti-cancer immune cells, suppressed immune checkpoint regulation, and modulated gut microbiota, contributing to their anti-cancer effects. In the context of ovarian cancer, the antioxidant effects of GLPs and GLE have been elucidated, underscoring their potential as therapeutic agents. GLPs were found to enhance antioxidant enzyme activity and promote the detoxification of harmful agents, highlighting their role in modulating the cellular antioxidant response.

The collective findings of these studies suggest that GLPs hold immense promise for cancer management, offering both direct anti-cancer effects and the potential to enhance existing cancer therapies. These effects encompass a wide range of mechanisms, from apoptosis induction to immune system modulation and anti-inflammatory activities. Additionally, the antioxidant properties of GLPs open up new avenues for research into the development of novel treatments for ovarian cancer. While the studies discussed here provide valuable insights into the potential clinical applications of GLPs, it is imperative to acknowledge that further research is needed to translate these findings into effective clinical interventions. Future studies should focus on the optimization of GLP-based therapies, including dosage, administration methods, and potential side effects.

## Clinical Studies on GLPs and immune function in cancer patients

Until now, a limited number of clinical trials have exclusively investigated the anti-cancer properties of GLPs. Most studies have explored GLPs in combination therapy with other natural or synthetic chemotherapeutic agents. These clinical studies have revealed promising insights into the potential of GLPs to enhance the immune system in patients with advanced solid tumors.

In a double-blind, placebo-controlled clinical trial, Gao, Dai et al. [26] assessed the safety and efficacy of Ganopoly^®^, a specific type of GLPs, in patients with advanced lung cancer. Sixty-eight patients with histologically confirmed advanced lung cancer received Ganopoly^®^ or a placebo at a dosage of 600 mg, three times daily for 12 weeks. The results indicated that Ganopoly+ significantly improved immune system functions, including increased levels of total T cells, NK cells, and an improved ratio of CD4 to CD8. Moreover, patients reported an enhanced quality of life based on Karnofsky score.

In another study, Gao, Zhou et al. [26, 27] explored the effects of Ganopoly® on immune functions in 44 patients with advanced-stage cancer. These patients were treated with 1800 mg of Ganopoly® thrice daily for 12 weeks. The findings demonstrated that Ganoderma treatment substantially elevated the levels of immune-related factors, including IL-2, IL-6, and IFN-γ while reducing the levels of IL-1 and TNF-α. The number of CD56 + cells significantly increased, and there were marginal increases in CD3 + , CD4 + , and CD8 + cells, with no significant change in the CD4:CD8 ratio. Moreover, after 12 weeks of Ganopoly treatment, enhanced responses to PHA and improved natural killer cell activities were observed in most patients, suggesting the potential of Ganopoly to boost immune activities in advanced-stage cancer cases.

In a study conducted by Goino [30], a hot water extract of *G. lucidum* (150 mL, three times a day, 35 days) in combination with Panax ginseng and Coriolus versicolor, was administered to 46 patients at advanced stages of various cancer types. This therapeutic approach improved appetite and sleep quality and reduced stress in 50% of the patients, including those with breast, cervical, cutaneous, lung, and ovarian cancers.

Furthermore, a clinical trial by Chen, Hu et al. [13] involved 74 advanced colorectal cancer patients who received oral GLPs at a dose of 5.4 g/day for 12 weeks. In 41 patients, GLPs were found to increase the number of CD3, CD4, CD8, and CD56 lymphocytes, as well as levels of IL-2, IL-6, and IFN-γ. The treatment also enhanced natural killer cell activities and the mitogenic reactivity to PHA, while reducing levels of IL-1 and TNF-α. Although no statistical changes were observed in three patients, these findings suggest that GLPs may have immune-stimulating effects in advanced colorectal cancer patients.

In a study by Shing, Leung et al. [101], treatment with *G. lucidium* capsules for six months increased mitogen-stimulated lymphoproliferation effects in immunocompromised children bearing leukemia. Moreover, Oka, Tanaka et al. [79] conducted a controlled trial involving colorectal cancer patients who received *G. lucidium* water extract for 12 months. The results showed a significant decrease in both the number and size of adenomas compared to the control group that received no treatment. For breast cancer patients undergoing endocrine therapy, the administration of spore powder of *G. lucidium* was shown to significantly enhance cancer-associated fatigue and improve quality of life, as reported by Zhao, Zhang et al. [138].

Suprasert, Apichartpiyakul et al. [111] conducted a randomized, double-blinded study to investigate the efficacy of *G. lucidium* extract in treating gynecologic cancers. In this study, 60 individuals with gynecologic cancer, who had previously failed chemotherapy regimens at least twice, were randomly assigned to three groups: placebo, *G. lucidium* water extract (6000 mg/day), and *G. lucidium* spore (6000 mg/day). The 12-week treatment was assessed for its impact on immunomodulation, toxicity, and quality of life. While approximately half of the patients were excluded from the study due to disease progression, the best response to treatment was disease stability, with rates of 38.1% in the water extract group and 50% in the spore group, while the placebo group showed no disease stability. The one-year survival rates were 63.6% for the water extract group, 60% for the spore group, and 44% for the placebo group. No significant alterations in hematological and non-hematological parameters, quality of life, or immune system functions were observed among the patients in the three groups. This study suggests that *G. lucidium*, whether as a water extract or spore, may help control gynecologic cancers in salvage settings without adverse effects.

The clinical studies examining the effects of GLPs on immune function in cancer patients offer valuable insights into the potential of GLPs as an adjunct to cancer therapies. These trials provide compelling evidence that GLPs may enhance immune responses and quality of life in cancer patients, albeit some key considerations should be addressed.

First, it is worth noting that these clinical studies primarily involved patients with advanced-stage cancer. While the results demonstrate improvements in immune parameters, it is essential to consider whether GLPs could also be beneficial in the earlier stages of cancer, potentially for preventive or adjuvant purposes. Expanding research to include patients across different stages of cancer could provide a more comprehensive understanding of GLPs' potential in cancer management. Second, the various administration methods and dosages across the studies raises questions about the optimal way to deliver GLPs for cancer patients. Understanding the most effective dosages, duration of treatment, and delivery mechanisms is crucial for maximizing the benefits and minimizing any potential side effects. Future studies should aim to establish standardized protocols for administering GLPs in clinical settings. Moreover, while the studies demonstrate improved immune parameters, including increased levels of T cells, NK cells, and altered cytokine profiles, a comprehensive assessment of the long-term effects and potential side effects of GLP treatments is warranted. This is particularly important when considering the use of GLPs alongside other chemotherapeutic agents. A detailed evaluation of the safety profile and potential interactions with other treatments is vital for clinical practice. A noteworthy aspect of these studies is the potential of GLPs to alleviate cancer-related symptoms and improve the quality of life. These findings are valuable and suggest that GLPs may provide holistic benefits beyond their immune-modulating effects. Future research should focus on identifying the specific mechanisms responsible for these quality-of-life improvements and whether they apply to various cancer types.

In terms of future prospects, these clinical studies open doors to further investigations into the use of GLPs in various cancer types and stages. More extensive randomized controlled trials, including larger patient populations and diverse cancer types, could provide a broader understanding of the applications of GLPs in clinical oncology.

Thus, the clinical studies focusing on GLPs and immune function in cancer patients demonstrate the potential benefits of GLPs as adjuncts to cancer therapies. These findings call for further exploration of GLPs in different stages of cancer, standardized protocols for administration, long-term safety assessments, and mechanisms behind quality-of-life improvements. As we move forward, understanding the broader implications of GLPs in cancer treatment will be crucial for harnessing their full potential and offering improved care for cancer patients.

## Application of *G. lucidum* polysaccharides as nanoparticle delivery system

Nanoparticles (NPs) are extremely small particles with diameters ranging from 10 to 1000 nm. Using NPs as delivery systems offers several advantages, including prolonging the half-life of drugs, enhancing the solubility of hydrophobic medications, and controlling or sustaining drug release. Various types of NPs, have demonstrated promise as targeted delivery systems. These include organic carriers like lipids (liposomes and solid lipid nanoparticles), polymers (dendrimers, micelles, and polymeric nanoparticles), virus-based nanoparticles, as well as inorganic carriers like carbon nanotubes and mesoporous silica nanoparticles.

Natural polysaccharides-based NPs owning to their particular structures are appropriate candidates for encapsulation bioactive ingredients. Additionally, polysaccharides-based NPs enjoy good bioavailability, biocompatibility, biodegradability, low toxicity, and side effects for the delivery of active components. Polymer NPs represent optimal drug delivery vehicles in cancer therapy, mitigating the limitations associated with anticancer drugs, including poor water solubility, elevated toxicity, and undesirable side effects. Nonetheless, it is important to note that the majority of polymer-based NPs lack intrinsic anticancer properties.

### Studies on GLPs-based NP delivery systems

As mentioned previously, GLPs demonstrate remarkable anti-cancer and immunoregulatory features. Thus, its application as a NPs carrier improved the health benefits of core materials. GLPs have been employed to deliver several bioactive components to the particular body site, control release, inhibit fast cellular metabolism and clearance, and enhance their biological attributes. Thus far, there have been limited studies on GLPs-based NP delivery systems, and a summary of these studies is presented in Table 3. In this part, some studies related to applying GLPs as a nanocarrier delivery system have been mentioned.

**Table 3:** A brief review of the application of GLP-based nanoparticles in cancer treatment

Drug	Particle size (nm)	Preparation method	Applied Cell type/Animal	Entrapment Efficacy (%)	Results	Refs.
CS-GLPs	217 ± 6	ion-revulsion	HepG2, HeLa and A549 cell lines (0- 6 μg/mL, 24 h)	25.01	The NPs containing GLP exhibited more pronounced tumor suppression and enhanced growth-promoting effects compared to the free GLP solution	[60]
SeNP-SGLP	25	Chemical resuction and dispersion	Raw 264.7 cell lines (0–100 μg/mL, 0–24 h)	–	The anti-inflammatory properties of SeNPs-GLPS may play a role in the ongoing efforts to combat cancer and inflammation	[115]
GLPs microemulsion	87.94 ± 3.17	queous titration method	A549 and Caco-2 cell lines (0–1000 μg/mL of oil phase, 0–24 h); Xenograft mice (8 mg/kg)	–	This study elucidated the potential mechanism of the spatial relationship between GLps and microemulsion and confirms the importance of GLP in tumor accumulation and its anti-tumor effectiveness	[34]
GLP-BiNP	10 ± 3	electrostatic interaction between sulfhydrated GLP and BiNP	CD40, CD80, CD86, and MHCII cell lines (0–80 μg/mL, 0–24 h); Xenograft mice (2.5 g/kg/day, 14 days)	–	GLPBiNP might offer an additional anti-inflammatory effect to counteract any potential toxicity resulting from the bismuth metal in the nanoparticles	[132]
rGO-Fe3O4-GL-PF	11.2 ± 4.8	Thermal process and oxidation and chemical absorption	A549 cell lines (0.1–150 μg/mL, 24 h)	11	NPs held great promise to develop specialized drug delivery systems for cancer therapy	[54]
GLP-RCPBA-DPA-DHA-HCPT	98.49 ± 5.16	Nanoprecipitation	MCF-7 cell lines (0.7 μg/mL, 0–72 h); xenograft mice (10 mg/kg/2 days, 8 days)	28.34	The formulated nanoparticles effectively eliminate cancer cells, impede tumor progression, and result in minimal side effects	[142]
Gold NPs	38.3 ± 0.3	Chemical reduction	CD40, CD80, CD86, and MHCII cell lines (0–40 μg/mL, 0–24 h); Xenograft mice (30 mg/kg/4 days, 12 days)	–	GLPs have the potential to be integrated into nanocomposites with immunoregulatory properties, thereby improving their effectiveness in cancer therapy	[137]
Gold NPs	25–29	Chemical reduction	HT-29 cell lines	–	The apoptotic effects on cancer cells were found to increase in a dose-dependent manner when treated with Gold NPs	[21]
EC-GLT-PVA-GLPs	221–253	layer-by-layer electrospinning	SGC-7901, A549, Hela and Caco-2 cell lines	7.9	This nanomedical film exhibited a promising antitumor effect at the cellular level	[71]
APBA − MTX/HCPT-GLP	191 nm	Nanoprecipitation	MCF-7 cell lines xenograft mice	21.5	In vivo, the GLP-APBA-MTX/HCPT nanoparticles demonstrated more potent tumor-inhibiting effects with fewer associated side effects than free MTX and HCPT	[141]

CS: Chitosan; Se (Selenium); Bi (Bismuth Sulfide); rGO-Fe3O4-GLE-PF (Graphene oxide-magnetite-*ganoderma lucidum* extract- Pluronic F-127); GLP-RCPBA-DPA-DHA-HCPT (*G. lucidum* polysaccharides-rutin-carboxyphenyl boronic acid- dithiodipropionic acid- dihydroartemisinin-10-hydroxy camptothecin); EC-GLT-PVA-GLP (ethycellulose-*G. lucidum* triterpenes-polyvinyl alcohol-*G. lucidum* polysaccharides); APBA − MTX/HCPT-GLP (3-aminophenylboronic acid-methotrexate/10-hydroxycamptothecin-*G. lucidum* polysaccharides)

#### Chitosan-based NPs loaded with GLPs

In a research conducted by Li, Hu et al. [60], innovative chitosan-based NPs loased GLPs were prepared and assessed for their physical and chemical characteristics, as well as their anti-tumor activity. The study involved in vitro cytotoxicity investigations using HepG2, HeLa, and A549 cancer cell lines and an assessment of their capacity to stimulate the growth of mouse spleen cells. The results revealed that chitosan NPs loaded with GLPs exhibited notably higher cytotoxicity against tumor cells and a more remarkable ability to promote the development of mouse spleen cells compared to empty chitosan NPs. These GLPs-NPs demonstrated substantial anti-tumor efficacy, positioning them as promising candidates for clinical applications.

#### GLPs in Coix Oil-based microemulsion

In a study by Guo, Yuan et al. [34], GLPs were incorporated into a coix oil-based microemulsion, and their effect on lung cancer cells was evaluated. The results showed that treatment of A549 cell lines with microemulsion containing GLPs (0–1000 μg/mL, 24 h) reduced the cell viability significantly in a dose-dependent manner. Furthermore, intragastrical administration of xenograft athymic nude BALB/c mice bearing lung cancer with GLPs microemulsion (2.5 g/kg/day, 14 days) reduced tumor weight and increased the immune index of serum.

#### Selenium NPs decorated with sulfate-derivative GLPs

In a study on inorganic NPs, Wang, Zhang et al. [115] utilized a one-step process to synthesize selenium nanoparticles (SeNPs) decorated with a sulfate derivative of GLPs (SGLPs). The resulting SeNPs- SGLPs complexes were stable, with a uniform diameter of approximately 25 nm. The anti-inflammatory properties of SeNPs-SGLPs were evaluated in murine Raw 264.7 macrophage cells induced by GLPs. The SeNPs-SGLPs complexes significantly reduced LPS-induced nitric oxide (NO) production in Raw 264.7 macrophages. RT-PCR analysis revealed a dose-dependent down-regulation of mRNA gene expressions for pro-inflammatory cytokines, including inducible NO synthase (iNOS), IL-1, and TNF-α. Conversely, the anti-inflammatory cytokine IL-10 was notably increased. In the NF-κB signaling pathway, SeNPs-SGLPs complexes effectively inhibited the phosphorylation of Iκ-Bα. Similar results were observed in inhibiting the phosphorylation of JNK1/2 and p38 mitogen-activated protein kinase (MAPKs), while ERK1/2 MAPK appeared unaffected by SeNPs-SGLPs. These findings collectively suggest that SeNPs-SGLPs complexes exhibit anti-inflammatory potential by modulating the secretion profiles of pro- and anti-inflammatory cytokines. This mechanism is partially attributed to inhibiting NF-κB, JNK1/2, and p38 MAPKs activation.

#### Bismuth sulfide NPs loaded with immunoreactive GLPs

Traditional radiotherapy may face challenges due to radiation-stimulated immunosuppression, necessitating alternative approaches for effective cancer treatment. A study presented a novel strategy involving Bismuth Sulfide nanoparticles (BiNP) loaded with immunoreactive GLPs in this context. This approach aimed to address the issue of reversing immunosuppression within the tumor microenvironment. To assess its effectiveness, 4T1 cells were subjected to X-ray radiation (4 Gy) followed by treatment with BiNP-GLPs at various concentrations (0–160 μg/mL) for 24 h. The results were compelling, with a significant reduction in the number and size of 4T1 cells compared to X-ray radiation alone. This effect could be attributed to the effective absorption of X-ray radiation by the bismuth element present in BiNP-GLPs, indicating their potential as radiosensitizers for cancer therapy. Furthermore, BiNP-GLPs were shown to activate dendritic cells effectively, as evidenced by the elevation of acid phosphatase activity, cytokine release, phenotype maturation markers, and T cell proliferation in the co-culture of dendritic cells (DC) and T cells. In xenograft mice bearing breast cancer, co-treatment with BiNP-GLPs (administered at 8 mg/kg) and X-ray radiation (4 Gy) significantly reduced tumor growth, apoptosis induction, and inhibition of lung metastasis. From a molecular perspective, BiNP-GLPs were found to alter tumor immunosuppression by increasing the proliferation of intratumor CD8 + cells and enhancing immune balance through an increase in the serum INF-γ/IL-4 ratio. These findings highlight the potential of BiNP-GLPs to suppress tumor growth through immune activation and radiosensitization, offering a promising avenue for cancer therapy [132].

#### Gold Nanocomposites-Based GLPs (Au-GLPs)

As mentioned earlier, natural polysaccharides like GLPs possess immunoregulatory properties, but their effectiveness in cancer treatment has been inconsistent, possibly due to low prescribed doses and rapid clearance in clinical applications. In order to evaluate the immunomodulatory responses of GLPs, Zhang, Pang et al. [137] fabricated gold nanocomposites-based GLPs (Au-GLPs). Au-GLPs efficiently activated dendritic cells (DCs). This activation was evident from the increased expression of CD80, CD86, CD40, and MHCII, reduced phagocytic ability and acid phosphatase activity, and enhanced cytokine transcription. Au-GLPs significantly stimulated the proliferation of CD4 + and CD8 + T cells in splenocytes. Co-culture experiments with DCs and T cells demonstrated that GLP-Au directly led to T cell proliferation. Combination therapy of female Balb/c mice bearing 4T1 breast cancer tumors with Dox (4 mg/kg) and Au-GLPs (30 mg/kg/every four days, 12 days) significantly reduced the tumor weight. Furthermore, Dox treatment promoted weight loss, presenting severe systemic toxicity in mice, which is prevalent for chemotherapeutic agents. However, co-treatment of mice bearing breast cancer with Au-GLP and Dox declined the rate of body weight loss induced by Dox. Additionally, Au-GLP and Dox co-treatment remarkably reduced pulmonary metastasis compared to Dox alone, indicating the anti-metastasis and growth inhibitory impact of the combination therapy. Also, treatment of xenograft mice with Dox alone decreased the number of CD4^+^ and CD8^+^ T lymphocytes. In contrast, co-treatment with GLP-Au and Dox increased the proliferation of CD4^+^ and CD8^+^ T cells, suggesting the immunomodulation effects of GLP-Au nanoparticles. Therefore, GLPs may be designed for NPs delivery systems to improve immunoregulation, suppress tumor propagation, and enhance their efficacy in tumor therapy.

#### rGO-Fe3O4-GL-PF for controlled drug delivery

In a separate investigation, a superparamagnetic nanocomposite composed of graphene and magnetite (rGO-Fe3O4) was created using a straightforward in-situ chemical method. This versatile rGO-Fe3O4 nanocomposite was developed to serve as a drug carrier that can be guided by external magnetic fields, enabling targeted drug delivery for cancer treatment. GLE was harnessed to stabilize the rGO-Fe3O4, enhancing its water dispersibility and stability through hydrogen bonding. Additionally, Pluronic F-127 (PF) was introduced to mitigate overall cytotoxicity. To investigate the potential of rGO-Fe3O4-GL-PF for controlled drug delivery, quercetin, a naturally occurring polyphenolic flavonoid with anticancer properties, was utilized. In vitro cytotoxicity assessments revealed significant cytotoxicity against A549 cells, underscoring the potential of GLE as a targeted drug delivery carrier for cancer therapy [54].

#### pH and redox-responsive drug delivery with RCGDDH NPs

Zheng, Zhao et al. [142] introduced an innovative polymeric NP system based on GLPs, known as RCGDDH NPs, which exhibit dual responsiveness to changes in pH and redox conditions. This NP platform efficiently delivers these therapeutic agents: rutin-carboxyphenyl boronic acid (CPBA), dithiodipropionic acid (DPA), dihydroartemisinin (DHA), and 10-hydroxy camptothecin (HCPT). The encapsulated drugs are released in a programmed manner: rutin is released in the slightly acidic tumor microenvironment, while DHA and HCPT are released in the reductive redox environment within tumor cells. Significantly, GLPs, acting as the carrier, enhance drug delivery and exhibit inherent anticancer activity. The RCGDDH NPs are finely sized at approximately 98 nm. In vitro release studies revealed that, under pH 5.2 and 10 mM reductive glutathione, the release of HCPT and DHA from the nanoparticles increased by 2.5 and 2.7 times, respectively, compared to normal physiological conditions. The released rutin in the nanoparticles also demonstrated a pronounced inhibitory effect on matrix metalloproteinase MMP-9. Further in vitro and in vivo experiments demonstrated the efficacy of RCGDDH NPs in eradicating tumor cells, inhibiting tumor growth, and minimizing side effects. These results collectively indicate that the pH and redox dual-responsive RCGDDH NPs hold significant promise as a therapeutic candidate for treating tumors.

#### Co-Delivery of bioactive components using GLPs-Based NPs

Zheng, Zhao et al. [141] applied GLPs-NPs as a dual pH and redox-responsive carrier agent to deliver three bioactive components, including rutin-carboxyphenyl boronic acid (CPBA)-GLP-dithiodipropionic acid (DPA)-dihydroartemisinin (DHA)/10-hydroxy camptothecin (HCPT). CPBA-DPA-DHA- HCPT-GLPs NPs reduced the viability of tumor cells in vitro and in vivo with lower side effects. In a study, GLPs-based NPs were fabricated to co-deliver 10- hydroxycamptothecin (HCPT) and methotrexate (MTX). Administration of xenograft mice bearing breast cancer with GLPs-HCTP-MTX NPs (10 mg/kg/ every two days for eight days) reduced tumor growth and increased cancer suppressive results with lower adverse effects [141].

#### Sustained release for an antitumor composite

Sustained release approaches offer a solution by prolonging the duration of effective concentration while reducing the peak concentration time. In this study, Lu, Li et al. [71] developed a sandwich-structured antitumor composite consisting of a three-layer film, which includes Top and bottom layers of ethyl cellulose (EC) containing *G. lucidum* triterpenes (GLT) and a middle layer of polyvinyl alcohol (PVA) containing GLPs. These films were constructed using layer-by-layer electrospinning to achieve sustained release of the antitumor activity of GLT and GLP for cancer therapy. This sandwich-structured material exhibits the potential for sustained antitumor release, high loading capacity (1.6% for GLT and 7.9% for GLP), and a large surface area. The study encompassed the characterization of morphology, structure, biocompatibility, and the release profile of encapsulated GLP and GLT in this composite. In vitro antitumor effects of this medicated film were investigated using carcinoma cells, including SGC-7901, A549, Hela, and Caco-2, with resulting IC50 values of 51.2, 90.7, 93.0, and 21.7 μg/mL, respectively. In summary, this medical film demonstrated effective antitumor activity at the cellular level.

### The versatility and mechanisms of GLPs in various NP delivery platforms

One notable aspect of these studies is the diverse range of NPs used in conjunction with GLPs, including chitosan-based NPs, microemulsions, selenium nanoparticles, superparamagnetic nanocomposites, and polymeric NP systems. This diversity highlights the adaptability and versatility of GLPs in various drug delivery platforms, allowing for tailored approaches to different therapeutic needs. However, it is essential to recognize that while GLPs enhance the efficacy and safety of these NPs, the mechanisms behind this enhancement still require in-depth elucidation. Further research should delve into the interactions between GLPs and these different NP types, shedding light on the underlying principles governing their synergistic effects.

The studies demonstrate that GLPs can enhance the anti-tumor activity of these NPs. For instance, the chitosan-based NPs loaded with GLPs exhibited significantly higher cytotoxicity against tumor cells, offering a compelling argument for their potential application in cancer therapy. Nevertheless, the detailed molecular pathways through which GLPs potentiate the anti-tumor effects of these NPs remain somewhat elusive. Investigating the specific signaling pathways and immune responses triggered by the combination of GLPs and NPs can provide a deeper understanding of their mechanisms of action.

The controlled release features of these NP systems are a valuable asset for drug delivery. By encapsulating bioactive agents in GLPs-based NPs, the studies illustrate how the release of these agents can be programmed to match the needs of the tumor microenvironment, significantly enhancing the precision and effectiveness of cancer treatment. Future investigations should further refine these NP systems to optimize drug release profiles, considering factors such as pH, redox conditions, and the specific characteristics of the tumor environment.

### Challenge and promising Future for GLPs-based NP systems

Despite the promising results, it is crucial to acknowledge that clinical applications of these GLPs-based NP systems are still in their infancy. For instance, while the studies demonstrate the anti-tumor efficacy of these systems in vitro and in vivo, there is a need for more comprehensive preclinical and clinical trials to assess their safety and effectiveness in human patients. Addressing potential challenges related to biocompatibility, stability, and long-term safety is essential.

Therefore, the studies involving GLPs as NP delivery systems hold substantial promise for future research and clinical applications in the fields of cancer therapy, immunomodulation, and drug delivery. These critical analyses highlight the need for further investigations into the molecular mechanisms of GLP-NP interactions, the specific pathways responsible for their enhanced efficacy, and the refinement of controlled drug release profiles. As these findings continue to evolve, GLPs-based NP systems will likely play an increasingly vital role in developing innovative and effective treatments for various diseases. Nonetheless, rigorous testing and validation are essential to ensure their safety and efficacy in clinical practice. Future studies should aim to bridge these gaps in knowledge and bring us closer to realizing the full potential of GLPs in NP-based drug delivery systems.

## GLPs toxicity

The immunology and toxicology of Ganoderma have been considered in various studies. It has been reported that oral administration of *G.lucidium* hot water extract to mice (5000 mg/kg/day, 30 days) indicated no adverse effects on body weight, organ features, and hematological factors. Additionally, the polysaccharide fraction of *G.lucidium* hot water extract at the same dose generated no lethal or adverse impacts. Oral administration of *G.lucidium* (10 g/kg) in ovariectomized mice showed no severe effects and toxicity in the oestrus cycle. Taking *G.lucidium* syrup by rabbit (4–140 mL/kg) and dogs (2–4 mL/kg) for ten days showed no chronic or acute toxicity [3].

Cisplatin and doxorubicin are among the most common chemotherapeutic agents, but they disclose dose-dependent nephrotoxicity and cardiotoxicity, respectively. It has been revealed that cisplatin reduced CAT, GSH-Px, and SOD and increased creatinine. However, co-treatment of GLPs and cisplatin elevated antioxidant enzymes to their normal level and decreased urea and serum creatinine and lipid peroxidation stimulated by cisplatin. Similarly, co-treatment of GLPs and doxorubicin of cancer cells reduced cardiotoxicity, lipid peroxidation, and creatinine kinase while significantly improving antioxidant enzymes CAT, GSH, and GPx activity. Hence, GLPs are safe anti-tumor agents that reduce the cytotoxicity induced by chemotherapeutic compounds and can be applied along with chemical anti-cancer agents as adjuvant therapy to attenuate their side effects [1, 97, 98]. However, consumption of GLPs by patients under treatment with anti-diabetics or anti-coagulants should be carefully considered due to the anti-coagulant and blood glucose-reducing effects of GLPs.

## Conclusion

In recent years, the global interest in exploring the health benefits of edible herbal medicines has been on the rise. One such herbal remedy that has garnered considerable attention is *G. lucidum*, a medicinal mushroom renowned for its rich reservoir of therapeutic and pharmaceutical compounds. Among its numerous attributes, *G. lucidum* stands out for its remarkable anti-cancer properties, making it a subject of intense research and clinical investigation. Central to its arsenal of beneficial components are GLPs, which play a pivotal role in conferring a wide array of biological and medicinal benefits.

The primary objective of this study was to delve into the anti-cancer activities of GLPs, shedding light on their potential to ameliorate the toxicity associated with chemotherapy and their ability to enhance targeted drug delivery. The findings unveiled within these pages resonate with optimism, revealing that GLPs wield potent anti-cancer effects through an impressive repertoire of mechanisms. These mechanisms encompass their capacity for cytotoxicity, role as antioxidants, ability to induce apoptosis, involvement in ROS generation, and antiproliferative effects. It is evident that GLPs offer a multifaceted approach in the fight against cancer, serving as formidable allies in the quest for effective and less toxic treatments.

The quest for advanced cancer therapies has led to an exploration of innovative delivery systems, and in this context, GLPs have not been left behind. The study embarked on a journey to explore the potential of GLPs-based NPs as delivery vehicles for bioactive constituents. These specially designed GLPs-based NPs promise to target various cancer tissues, thereby amplifying the biological activity of encapsulated compounds. This novel approach demonstrates the adaptability of GLPs and their potential as a tool for inhibiting cancer progression and, notably, for mitigating chemotherapy-related side effects.

The implications of this research extend beyond laboratory findings and theoretical constructs. The anti-cancer prowess of GLPs, as validated through this study, represents a ray of hope for individuals and families touched by the burden of cancer. It is an acknowledgment of the potential of natural remedies to offer tangible support in the fight against one of humanity's most formidable adversaries. Furthermore, applying GLPs in combination therapies, as a natural adjunct to established treatment modalities, marks a significant milestone in pursuing holistic and patient-centered cancer care.

As we stand at the intersection of traditional wisdom and modern science, the GLPs emerge as a bridge between the past and the future of cancer treatment. The promise they hold as catalysts for progress in the field of oncology is tantalizing. Their potential to enhance the lives of cancer patients and the quality of cancer care encourages further exploration and fuels the conviction that the secrets of nature may hold the keys to a future where cancer is met with greater success and less suffering. In closing, the results presented here underscore the significance of GLPs and beckon researchers, clinicians, and patients alike to embrace their potential as formidable contenders in the fight against cancer.

## Data Availability

None.

## Abbreviations

Bax: Bcl-2-associated X protein
Bcl-2: B-cell lymphoma 2
CAT: Catalase
CDK2: Cyclin-dependent kinase 2
COX-2: Cyclooxygenase-2
CTLA-4: Cytotoxic T-lymphocyte–associated antigen 4
DJ1: Deglycase DJ-1
Dox: Doxorubicin
EGFR: Epidermal growth factor receptor
EMT: Epithelial-to-mesenchymal transition
ERK: Extracellular signal-regulated kinase
FAK: Focal adhesion kinase
GLPs: *Ganoderma lucidum* Polysaccharide
GSH-Px: Glutathione peroxidase
GSTP1: Glutathione S-transferase P1
IL: Interleukin
INF- γ: Interferon γ
iNOS: Inducible nitric oxide synthase
JAK: Janus kinase
JNK: C-Jun N-terminal kinases
LC3: Microtubule-associated protein 1A/1B-light chain 3
LPS: Lipopolysaccharide
MAPK: Mitogen-activated protein kinase
MNNG: N-methyl-N9-nitro-Nnitrosoguanidine
NAG-1: Non-steroidal anti-inflammatory drug-activated gene-1
NK: Natural killer
NQO1: NAD(P)H:Quinone oxidoreductase 1
Nrf2: Nuclear factor erythroid 2-related factor 2
PARP: Poly (ADP-ribose) polymerase
PD-1: Programmed Cell Death Protein 1
PHA: Phytohemagglutinin
PRMT6: Protein arginine methyl- transferase 6
RSGLP: Sporoderm‑removed spores *Ganoderma lucidum* polysaccharide
SOD: Superoxide dismutase
SRC: Steroid receptor coactivator
STAT5: Signal transducer and activator of transcription 5;
Teff: Effector T cell
TGFβR: Transforming growth factor beta receptor
TIM3: T cell immunoglobulin and mucin domain-containing protein 3
TNF-α: Tumor necrosis factor-α
Treg: Regulatory T cell
Trx: Thioredoxin
