# Correction: Exploring the anti-cancer potential of *Ganoderma Lucidum* polysaccharides (GLPs) and their versatile role in enhancing drug delivery systems: a multifaceted approach to combat cancer

**DOI:** 10.1186/s12935-024-03284-7

**Published:** 2024-05-22

**Authors:** Xiaoli Gao, Mina Homayoonfal

**Affiliations:** 1 Department of Life Science, Lyuliang University, Lyuliang, Shanxi 033001 China; 2 Research Center for Biochemistry and Nutrition in Metabolic Diseases, Institute for Basic Sciences, Kashan University of Medical Sciences, Kashan, Iran

**Correction to: Cancer Cell International (2023) 23:324**

10.1186/s12935-023-03146-8

In this article [1], there is an error in the email address provided by one of our co-authors. The correct email address for **Xiaoli Gao** is [**xief006@163.com**].

## References

[1] Gao X., Homayoonfal M. Exploring the anti-cancer potential of Ganoderma lucidum polysaccharides (GLPs) and their versatile role in enhancing drug delivery systems: a multifaceted approach to combat cancer. Cancer Cell Int. 2023;23, 324. 10.1186/s12935-023-03146-8 PMC10724890 38104078

